# Effectiveness of WeChat-Based Plus Scene-Graphics Health Education for Rehabilitation After Open Elbow Arthrolysis: Historical Control Study

**DOI:** 10.2196/58218

**Published:** 2025-10-21

**Authors:** Danling Fang, Yin Wang, Shiyang Yu, Wei Wang, Pingchen Lu

**Affiliations:** 1 Operation Room, Shanghai Sixth People's Hospital Affiliated to Shanghai Jiao Tong University School of Medicine, 600 YiShan Road, Shanghai, China, 86 021-3829-7726; 2 Department of Orthopedics, Shanghai Sixth People's Hospital Affiliated to Shanghai Jiao Tong University School of Medicine, Shanghai, China; 3 Department of Rehabilitation, Shanghai Jiao Tong University Affiliated Sixth People's Hospital, Shanghai, China

**Keywords:** elbow stiffness, open elbow arthrolysis, WeChat, scene graphics, range of motion, health education, rehabilitation, mobile health

## Abstract

**Background:**

Elbow stiffness often hinders daily tasks. Open elbow arthrolysis is effective but requires long-term postoperative rehabilitation. Traditional health education does not significantly improve patient cooperation or results. Handy and engaging tools such as WeChat and scene graphics may help.

**Objective:**

This study aims to assess the efficacy of WeChat-based health education combined with scene graphics following open elbow arthrolysis.

**Methods:**

This historical control study involved patients aged 18 years and older who underwent open elbow arthrolysis, had normal communication skills, and were proficient in using WeChat. Eligible patients were divided into 2 groups based on admission time: the control group (56 patients, enrolled from January to June 2021) and the WeChat group (56 patients, enrolled from July to December 2021). The control group had received traditional health education, whereas the WeChat group received health education using WeChat and scene graphics. Information in 4-part comics was shared through a WeChat public account. Patients accessed this account to receive daily lessons during hospitalization, followed by online instruction in a WeChat group after discharge until 12 weeks postoperatively. Outcome data were collected at 1, 6, and 12 weeks postoperatively. The primary outcome was elbow range of motion; secondary outcomes were elbow function, quality of life, and complication incidence.

**Results:**

The elbow flexion angle improved from 71.5° (SD 4.2°) to 124.2° (SD 11.7°) in the WeChat group and from 71.7° (SD 4.6°) to 114.4° (SD 13.6°) in the control group (difference 10.0°, 95% CI 4.9–15.1, *P*<.001). The mean elbow extension angle improved from 29.6° (SD 6.0°) to 6.4° (SD 2.5°) in the WeChat group and from 28.8° (SD 3.8°) to 10.1° (SD 3.4°) in the control group (difference –4.5°, 95% CI –6.5 to –2.5, *P*<.001). The mean forearm pronation angle improved from 31.9° (SD 4.0°) to 66.9° (SD 7.3°) in the WeChat group and from 33.0° (SD 4.2°) to 63.1° (SD 7.2°) in the control group (difference 4.9°, 95% CI 2.0-7.8, *P*=.001). The mean forearm supination angle improved from 30.2° (SD 3.7°) to 71.8° (SD 4.8°) in the WeChat group and from 30.4° (SD 4.1°) to 64.2° (SD 9.8°) in the control group (difference 7.7°, 95% CI 4.4-11.0, *P*<.001). The mean Mayo Elbow Performance Score increased from 58.0 (SD 3.7) to 80.4 (SD 5.7) in the WeChat group and from 58.9 (SD 2.8) to 75.8 (SD 6.9) in the control group (difference 5.5 points, 95% CI 2.8–8.2, *P*<.001). The mean 36-item Short Form Health Survey questionnaire score increased from 44.4 (SD 6.6) to 82.0 (SD 7.1) in the WeChat group and from 44.0 (SD 6.4) to 75.0 (SD 11.2) in the control group (difference 6.6 points, 95% CI 2.6-10.6, *P*=.002).

**Conclusions:**

WeChat-based health education combined with scene graphics was found to significantly improve elbow range of motion, elbow function, and quality of life.

## Introduction

Normal elbow mobility is fundamental in daily life. Elbow stiffness is a common condition, affecting up to 56% of individuals following elbow trauma [1,2]. It is characterized by an inability of the elbow to meet the patient’s needs for daily activities, work, and entertainment due to a limited range of motion (ROM) or rotation [3,4]. Open elbow arthrolysis (OEA) is the most common surgical intervention and has been shown to restore elbow function and ROM effectively [3,4]. The elbow can be released by removing bony impediments or scar tissue within the olecranon fossa, achieving intraoperative flexion greater than 130° and extension less than 10° [5,6]. However, postoperative rehabilitation is necessary for full elbow function recovery [4-8].

A growing body of evidence suggests that timely and proper postoperative rehabilitation leads to favorable outcomes. By contrast, inadequate rehabilitation severely hampers elbow recovery [4-8] and contributes to many postoperative complications, such as heterotopic ossification, new onset or exacerbation of ulnar nerve symptoms, and recurrent elbow stiffness [3,4,9,10]. According to incomplete statistics, the incidence rates of postoperative elbow issues, probability of renewed elbow stiffness, rate of secondary surgery, and rate of dissatisfaction are 56%‐87% [5], 8.4%‐47% [5,6], 10%‐34% [6,7], and 21% [7], respectively. Therefore, the precision of postoperative rehabilitation and patient adherence must be improved. However, achieving optimal postoperative rehabilitation is challenging due to its prolonged and intricate nature [6,11,12]. Standard postoperative rehabilitation lasts 1 year and is divided into 3 phases [6,13,14]. The first, acute phase begins on the first day and extends to 6 weeks after surgery; it involves passive, assisted, and active elbow flexion and extension exercises [6,13,14]. Rehabilitation starts with 30 movements on the first day, increasing by 30 daily until reaching 300 movements per day [6,13]. The second, subacute phase spans from 6 weeks to 3 months and requires maintaining 300 daily movements [13]. The third, functional phase lasts from 3 months to 1 year; during this phase, weight-bearing exercises are added, and intensity increases daily [13,14]. In addition to initial hospital-based rehabilitation, patients must continue their rehabilitation at home, making a comprehensive and accurate understanding of the relevant knowledge crucial for effective recovery [12,13,15]. However, with the increasing prevalence of enhanced recovery after surgery protocols [16,17], hospital stays have shortened, making traditional health education methods insufficient for patients to acquire adequate knowledge. Therefore, more efficient and feasible health education approaches are needed.

Telemedicine services have become increasingly popular for health education in recent years. Health care professionals use electronic information and communication technology to deliver and support health care services to patients, regardless of their location or transportation limitations [18]. WeChat is one of China’s most widely and frequently used free platforms [19-21] and offers convenient telemedicine services. It supports a variety of data types, including text, voice calls, videos, and images, enabling clear and accessible communication [20], which attracts many users. By the end of 2020, there were 1.2 billion monthly active users worldwide [21,22], many of whom actively seek online health care and health information exchange [23]. A previous survey revealed that one-third of participants regularly read health information articles on WeChat [24], and 98.53% used WeChat to search for health information [23]. The platform has been used in the health management of conditions such as public health literacy [25], cancer [26,27], asthma [28], chronic obstructive pulmonary disease [29], hypertension [30], myopia [31], coronary artery disease [32], and others. However, no studies have focused on its use for postoperative rehabilitation in patients with elbow stiffness.

Medical humanities is an interdisciplinary, multifaceted field that combines scientific knowledge and skills with respectful, compassionate care sensitive to patients’ and their families’ values, autonomy, and cultural backgrounds [33]. Medical comics are among the various medical humanities approaches that can potentially address challenging situations within medical settings [33,34]. They have been used to promote adolescent peer support and healthy sexual behaviors [34], raise awareness about diabetes [35], improve the quality of palliative care [36], and increase awareness of whole-body donation [37]. Scene-based graphics are an approach that simplifies knowledge points into comics and conveys messages through various humorous scenarios, making the information easily understandable and memorable. The graphics have transformed activities of daily living scales into comics for rapid and straightforward evaluation [38]. Additionally, interactive games have been incorporated to complement the corresponding knowledge [39]. These games can be designed based on key knowledge points and learner characteristics, encouraging participants to engage more actively in learning and apply their knowledge more effectively. However, this approach is still in its early stages and is rarely used in postoperative rehabilitation. This study was conducted as a historical control study to evaluate the efficacy of WeChat-based health education combined with scene graphics in the postoperative rehabilitation of OEA.

## Methods

### Study Design

A 2-armed historically controlled study was conducted at a tertiary hospital in Shanghai, China.

Patient characteristics between the 2 groups were carefully analyzed and matched to minimize potential selection biases related to time. Patients were stratified by age, gender, type of elbow injury, and severity of the condition. For each stratum, the participants admitted from January to June 2021 were included in the control group, whereas those admitted from July to December 2021 with similar characteristics were assigned to the WeChat group. The follow-up period ended in March 2022.

Both groups received identical intervention content for the same duration. A trained research nurse collected baseline data upon admission, and a research doctor collected outcome data at baseline and at the first, sixth, and twelfth weeks after surgery. To ensure consistency in data collection, both the research nurse and research doctor received standardized training, and a detailed data collection manual defining each variable and measurement method was developed.

### Ethical Considerations

This study was registered with the Chinese Clinical Trial Registry (ChiCTR2000036004) and approved by the Ethics Review Committee of Shanghai Sixth People’s Hospital (approval number 2020‐028). Patients were informed of the study’s details during enrollment, including its purpose, procedures, risks, and benefits. They were also told about the confidentiality and waiver agreements, which guaranteed data protection and the right to withdraw without affecting their treatment. After understanding these terms, patients provided written consent to participate (see Multimedia Appendix 1). This research conformed to the Consolidated Standards of Reporting Trials (CONSORT) checklist, with adherence details provided in Multimedia Appendix 2.

### Participant Recruitment

Upon admission, patients received recruitment notifications through leaflets. These notices, which included an introduction to the research and a contact number, were distributed by research nurses after standardized admission education in the orthopedic ward.

To minimize potential biases, the nurses were trained to follow a scripted message (eg, “Whether or not you participate in this study will not affect your treatment”). Patients interested in joining this study contacted the provider number and completed an expression-of-interest form electronically, which was collected by a neutral coordinator. They then observed a 24-hour cool-off period during which they could withdraw at any time. Patients who confirmed their willingness to participate were assigned to research nurses. Basic participant characteristics were collected by a trained surgeon-nurse team through face-to-face communication, physical examination, and elbow function assessment using validated tools.

Patients were eligible if they were older than 18 years, diagnosed with elbow stiffness(see Multimedia Appendix 1), had undergone OEA for the first time, were conscious (Glasgow Coma Scale score of 15), could communicate normally in Chinese (Mandarin), possessed a mobile device (eg, smartphone or tablet) with internet access, and were capable of using WeChat. Exclusion criteria included severe systemic diseases (eg, uncontrolled hypertension with blood pressure consistently above 180/110 mmHg, poorly controlled diabetes with HbA_1c_ [glycated hemoglobin] >9%, or active malignancy); surgery within the past 6 months; comorbidities such as severe rheumatoid arthritis or peripheral neuropathy that could interfere with elbow rehabilitation; and known allergies to medications or materials used during the surgical procedure.

### Sample Size

The pilot study’s elbow extension and flexion degrees at 6 weeks postsurgery were used to estimate the sample size [39]. Before applying the sample size calculation formula, a variance homogeneity test was performed for elbow flexion and extension angles in the 2 groups. With a test power (1 – β) of 80%, a significance level (α) of 5% (2-sided), a 1:1 group size ratio, and elbow flexion of 128.6° (SD 5.8°) and 119.4°(SD 9.8°), respectively (after confirming variance homogeneity), the required sample size was calculated as 24 patients. Similarly, for elbow extension angles of 14.3° (SD 11.3°) and 22.9° (SD 8.8°), respectively (after confirming variance homogeneity), 14 patients were required. Considering a 20% attrition rate, the adjusted minimum sample size was 29 participants per group. As patients’ responses to program participation were positive, 56 participants were ultimately included in each group.

### Blinding

No blinding was implemented in this study. The surgical team and research members were aware of group allocations, as the intervention required explicit instructions for the WeChat-based rehabilitation program. Patients were enrolled into different groups at varying admission times to minimize interaction, and all participants were informed about the study procedures without masking group details. Primary outcomes (eg, elbow ROM, Mayo Elbow Performance Score [MEPS]) were measured using standardized protocols with calibrated instruments to minimize subjective bias. All data were recorded electronically and analyzed by a statistician blinded to group allocations.

### Interventions

All participants underwent OEA performed by the same surgical team and received health education from the same interveners. The elbow rehabilitation knowledge was based on the book “The Elbow: Principles of Surgical Treatment and Rehabilitation” [14] and the research from Sun et al [6,13]. The control group received 12 weeks of traditional health education, while the WeChat group received 12 weeks of WeChat-based health education combined with scene graphics. Interventions were conducted from the first day up to 12 weeks after surgery. Typically, 3-5 patients were hospitalized simultaneously, and interventions started concurrently. Patients who were lost to follow-up or who changed treatment plans (ie, participated in another postoperative project that might have affected the results) were withdrawn from the intervention (see Multimedia Appendix 1).

### Control Group

The control group received a card with a QR code on the second day after admission. Patients were instructed to access an online manual titled “Elbow Rehabilitation” by scanning the code using WeChat (see Multimedia Appendix 1). They could study this manual independently from the second day of admission until 12 weeks postoperatively. Research nurses addressed patients’ questions during morning nursing care at 7:30 AM daily. Additionally, patients could ask questions during nursing sessions. The rehabilitation process was carried out 3 times a day, including 1 guided session and 2 independent sessions. It began with 30 exercise sets on the first day, increasing by 30 sets daily until reaching 300 sets per day. A rehabilitation therapist provided guidance every day at 9:00 AM for 30 minutes in the multimedia demonstration classroom, correcting any incorrect movements. For the other 2 independent sessions, research nurses regularly patrolled the exercise area and promptly corrected any movement inaccuracies they observed.

After discharge, weekly follow-up calls were made to ensure continuity of the recovery process. These calls involved checking the patient’s current condition, answering any questions about ongoing rehabilitation, and providing guidance based on their progress.

### WeChat Group

The multidisciplinary team comprised a surgeon, a nurse manager, a rehabilitation therapist, a platform maintenance specialist, and elbow joint nurse specialists who collaborated to develop the health education scheme. The surgeon oversaw the intervention, trained team members, and assessed patients’ elbow function. The nurse manager supervised the implementation of the intervention, while the rehabilitation therapist developed elbow rehabilitation content and instructed patients on the exercises. The 3 nurse specialists delivered the health education.

The health education materials used were the same as those provided to the control group. The research team then transformed these materials into a comic book. The process began with the team crafting a script and a set of questions for the comic, which were submitted to a surgeon for review. After discussions and refinements among the team members, the content was finalized. The research team then partnered with a cultural media company to convert the script into a visual comic format (see Multimedia Appendix 3). After completing the comic book, the team created a WeChat public account featuring the “Elbow Joint Zone,” which was designed and divided by platform maintenance specialists into 4 information modules based on the comic book (for content and module names, see Table S1 in Multimedia Appendix 1). The research team then discussed the offline class schedule and arranged the modules according to difficulty (see Table S2 in Multimedia Appendix 1). To further increase engagement, the team also created PowerPoint (Microsoft Corporation) presentations used in offline education through interactive games.

**Table 1.** Baseline characteristics of 112 adult patients with posttraumatic elbow stiffness who underwent open elbow arthrolysis, comparing the WeChat intervention group with a control group in a historical control study conducted at a tertiary first-class hospital in Shanghai, China, between January 2021 and March 2022.

Characteristics	Total (n=112)	WeChat group (n=56)	Control group (n=56)	*t* test (*df*)^a^/chi-square (*df*)/Mann-Whitney *U* test	*P* value
Age (years), mean (SD)	36.0 (3.1)	35.98 (3.4)	36.1 (2.9)	*0.121 (110)*	.90
Sex, n (%)				*0.895 (1)*	.34
Women	59 (52.7)	32 (57.1)	27 (48.2)		
Men	53 (47.3)	24 (42.9)	29 (51.8)		
Highest level of education, n (%)				*4.685 (4)*	.32
Primary school	2 (1.8)	1 (1.8)	1 (1.8)		
Junior high school	13 (11.6)	7 (12.5)	6 (10.7)		
Senior high school	26 (23.2)	12 (21.4)	14 (25.0)		
Junior college	44 (39.3)	18 (32.1)	26 (46.4)		
University degree or above	27 (24.1)	18 (32.1)	9 (16.1)		
BMI (kg/m^2^), mean (SD)	21.6 (1.81)	21.5 (1.8)	21.77 (1.78)	*0.793 (110)*	.43
Disease side, n (%)				*0.144 (1)*	.71
Left	60 (53.6)	31 (55.4)	29 (51.8)		
Right	52 (46.4)	25 (44.6)	27 (48.2)		
Duration of disease (months), median (25th percentile-75th percentile)	8.2 (7.4-8.8)	8.2 (7.5-8.8)	8.0 (7.2-8.8)	–0.136	.26
Duration of surgery (minutes), median (25th percentile-75th percentile)	125 (124-126)	125 (124-126)	125 (124-126)	–0.085	.93

a Independent sample *t* test values are in italics.

**Table 2.** Postoperative functional and quality-of-life outcomes, including elbow flexion/extension, forearm pronation/supination, MEPS^a^, and SF-36^b^ scores, were assessed preoperatively and at 1, 6, and 12 weeks postsurgery in 112 adult patients with posttraumatic elbow stiffness who underwent open elbow arthrolysis. This historical control study, conducted at a tertiary first-class hospital in Shanghai, China (January 2021 to March 2022), compared outcomes between a WeChat-based intervention group and a control group.

Outcomes	Total (n=112)	WeChat group (n=56)	Control group (n=56)	*t* test (*df*)	*P* value
Elbow flexion^c^ (degrees), mean (SD)					
Baseline	71.6 (4.4)	71.5 (4.2)	*71.7 (4.6)*	*0.236 (110)*	.81
Postoperative 1 week	84.6 (10.2)	86.0 (8.8)	83.3 (11.4)	*−1.392 (102.94)*	.17
Postoperative 6 weeks	101.3 (10.3)	103.0 (10.3)	99.6 (10.1)	*−1.773 (110)*	.08
Postoperative 12 weeks	119.3 (13.6)	124.2 (11.7)	114.4 (13.6)	*−4.063 (110)*	<.001
Elbow extension^d^ (degrees), mean (SD)					
Baseline	29.2 (5.0)	29.6 (6.0)	28.8 (3.8)	*−0.903 (92.79)*	.37
Postoperative 1 week	22.9 (6.1)	22.9 (7.0)	23.0 (5.2)	*0.061 (100.90)*	.95
Postoperative 6 weeks	14.7 (6.3)	13.7 (5.4)	15.7 (5.9)	*1.716 (110)*	.06
Postoperative 12 weeks	8.2 (3.5)	6.4 (2.5)	10.1 (3.4)	*6.427 (101.95)*	<.001
Forearm pronation^e^ (degrees), mean (SD)					
Baseline	32.5 (4.1)	31.9 (4.0)	33.0 (4.2)	*1.387 (110)*	.17
Postoperative 1 week	46.2 (7.6)	45.6 (7.2)	46.8 (8.0)	0.880 (110)	.38
Postoperative 6 weeks	58.9 (8.9)	60.9 (8.8)	56.8 (8.4)	*−2.505 (110)*	.01
Postoperative 12 weeks	65.0 (7.5)	66.9 (7.3)	63.1 (7.2)	*−2.794 (110)*	.006
Forearm supination^f^ (degrees), mean (SD)					
Baseline	30.3 (3.9)	30.2 (3.7)	30.4 (4.1)	*0.168 (110)*	.87
Postoperative 1 week	44.9 (8.6)	45.3 (8.4)	44.5 (8.9)	*−0.469 (110)*	.64
Postoperative 6 weeks	57.8 (10.0)	59.8 (10.0)	55.9 (9.8)	*−2.062 (110)*	.04
Postoperative 12 weeks	68.0 (8.6)	71.8 (4.8)	64.2 (9.8)	*−5.183 (80.03)*	<.001
MEPS^g^ scores, mean (SD)					
Baseline	58.5 (3.3)	58.0 (3.7)	58.9 (2.8)	*1.472 (110)*	.14
Postoperative 1 week	60.9 (4.8)	61.1 (6.5)	60.7 (2.1)	*−0.509 (66.70)*	.61
Postoperative 6 weeks	68.4 (4.8)	69.1 (5.2)	67.8 (4.2)	*−1.509 (110)*	.13
Postoperative 12 weeks	78.1 (6.7)	80.4 (5.7)	75.8 (6.9)	*−3.829 (110)*	<.001
SF-36^h^ scores, mean (SD)					
Baseline	44.2 (6.5)	44.4 (6.6)	44.0 (6.4)	*−0.334 (110)*	.74
Postoperative 1 week	55.0 (7.8)	55.3 (7.0)	54.7 (8.6)	*−0.399 (110)*	.69
Postoperative 6 weeks	70.3 (11.0)	71.0 (10.5)	69.7 (11.4)	*−0.619(110)*	.54
Postoperative 12 weeks	78.5 (10.0)	82.0 (7.1)	75.0 (11.2)	*−3.952(93.07)*	<.001

a MEPS: Mayo Elbow Performance Score.

b SF-36: 36-item Short Form Health Survey.

c Elbow.flexion: the normal range is 0°‐150°; the study target is 0°‐120°. A value closer to 150° indicates better function.

d Elbow extension: ideally, 0°‐5°; for this study, 0°‐10°. Higher values signal instability, reflecting poorer function.

e Forearm pronation: in this study, the target range was 50°‐70°, approaching the normal 70°‐85° range for better function.

f Forearm supination: the goal of this study is 50°‐70°; ideally, alignment with the normal range of 70°‐85° indicates better functionality.

g The scores range from 0 to 100 points, and are categorized as follows: excellent (90-100), good (75-89), fair (60-74), and poor (0‐59).

h This research employed physiological functioning as a metric, ranging from 0 to 100 points, with higher scores corresponding to enhanced quality of life.

On the second day of admission, the participants received the same card as the control group, featuring a QR code for the WeChat public account. They accessed the materials using the same procedure as the control group. The intervention consisted of 5 steps, as outlined below.

- Step 1 (Knowledge memory): The research nurse directed patients to access the Elbow Special Zone on the WeChat public account. Patients were instructed to dedicate fragmented time to self-studying the assigned knowledge until they had memorized it. They were encouraged to consult the research team for guidance if needed.
- Step 2 (Picture-based cloze test): The research nurse conducted a multimedia classroom session in the orthopedic ward using PowerPoint to display a comic with blank spaces that patients needed to fill in with the correct knowledge. Patients took turns answering and were praised for correct responses. For incorrect answers, the nurse provided detailed guidance. Patients who missed the session due to clinical examinations received face-to-face guidance.
- Step 3 (Error correction): The research nurse used a similar approach as in step 2. Patients were shown pictures with missing words related to high-frequency mistakes and asked to fill them in until they fully understood the content.
- Step 4 (Keypoint summary): The research nurse summarized key points and emphasized common problems discussed in step 2. These materials were compiled into a Word (Microsoft Corporation) document, printed for each patient, and the patients were urged to review them. The research team monitored learning progress through random questioning during daily ward rounds at 7:30 AM.
- Step 5 (Motion instruction): The rehabilitation therapist provided 1-on-1 instruction to teach proper movements and corrected mistakes until patients achieved the correct motions. Patients also learned independently via the WeChat public account.

Steps 2-5 were scheduled over 6 days, starting 1 day before surgery and continuing until 5 days after surgery (excluding the operation day). Sessions were held daily at 9:00 AM.

Before discharge, the research nurse created WeChat groups, each consisting of 3-5 patients, to ensure high-quality health education. The group names followed the format “group number + elbow care + operation date,” and patients used their name and telephone number as a unified identifier. On the day of discharge, the WeChat health education program was introduced to patients through 4 processes.

- Process 1 (Rehabilitation check-in): Every patient must submit a video in the WeChat group before 7:00 PM daily. The video should demonstrate all the motions the patient was required to complete that day and last no more than 5 minutes. The rehabilitation therapist reviewed the videos and corrected any incorrect movements. The @ function was used to remind patients who forgot to submit their videos.
- Process 2 (Knowledge consolidation): The research nurse used Questionnaire Star to design a “picture-based cloze test,” which was posted to the WeChat group at 8:00 AM daily. Patients were expected to submit their answers by 7:00 PM. The @ function was used as a reminder if necessary. Patients could also review the material via the WeChat public account.
- Process 3 (Error correction): The research nurse collected common error points and provided the correct answers in a Word document. Patients were reminded to review this document to improve their understanding.
- Process 4 (Answer questions and clarify doubts): Every evening from 7‐9 PM, the research nurse summarized key points and typical questions from process 2, and assessed the effectiveness of process 3 through random inquiries. Detailed answers were provided to any patient’s doubts.

### Measures

#### Primary Outcome Measures

Elbow ROM was measured using a handheld goniometer and included 4 angles: elbow flexion, extension, forearm pronation, and supination [40,41]. A surgeon performed 3 measurements for each angle using 3 landmarks—the lateral epicondyle, the tip of the acromion process, and the midpoint of the wrist—and the average ROM was calculated.

#### Secondary Outcome Measures

The secondary outcomes were elbow function, quality of life, and postoperative complications. (1) Elbow function was assessed using the MEPS, the most commonly used functional score [41]. MEPS comprises 4 subscales: pain (45%), ROM (20%), stability (10%), and daily function (25%). The total score ranges from 0 to 100 points and is classified into 4 categories: excellent (90-100), good (75-89), fair (60-74), and poor (0‐59) [10,40,41]. (2) Patient quality of life was evaluated using the 36-item Short Form Health Survey (SF-36) questionnaire [40]. The SF-36 is a self-rating scale consisting of 8 dimensions: physiological features, physiological function, somatic pain, general health, vitality, social function, affective function, and mental health. The Cronbach α coefficient ranges from 0.65 to 0.94. This study focused on the physiological function dimension. Scores range from 0 to 100, with higher scores indicating a better quality of life. (3) Postoperative complications included heterotopic ossification, new onset or exacerbation of ulnar nerve symptoms, and repeat elbow stiffness. Elbow heterotopic ossification was classified into 3 grades according to the Hastings and Graham classification: grade 1, no functional limitation; grade 2A, limited flexion and extension; grade 2B, limited pronation and supination; grade 2C, combination of 2A and 2B; and grade 3, ankylosis [1]. The new onset or exacerbation of ulnar nerve symptoms was assessed using the Dellon classification, which evaluates sensory symptoms (paresthesia, vibratory perception, and 2-point discrimination) and motor symptoms (muscle weakness and atrophy) [40,42]. Repeat elbow stiffness was diagnosed based on the elbow ROM, clinical presentation, and examination results [40,42].

### Data Collection and Analysis

All data were collected using a standardized data collection form and process. Demographic information, including age, sex, educational level, and BMI, as well as clinical data, such as operation duration, affected side, and disease duration, were obtained from medical records and patient interviews. A trained research nurse collected these data to facilitate comparison between the 2 groups. Primary and secondary outcome measures were recorded at baseline and at 1, 6, and 12 weeks postoperatively. The research surgeon collected the 1-week postoperative data during hospitalization, whereas data at 6 and 12 weeks were collected by the surgeon in the outpatient clinic. To ensure consistent follow-up, an automated reminder system, via SMS text messages or automated phone calls, similar to traditional phone calls, was used to remind patients in both groups to attend their reexamination. All data were analyzed by a statistician affiliated with the hospital’s clinical research center.

Data analysis was performed using SPSS Statistics version 26.0 (IBM Corp). The chi-square test was used to analyze the categories, including sex, disease side, and the incidence of postoperative complications. The *Z* test was used to analyze education levels. Independent sample *t* tests were used to analyze variables that satisfied the normality test, whereas nonparametric tests, specifically the Mann-Whitney *U* test, were applied for variables that did not meet the normality assumption.

The paired sample *t* test was applied to compare the preintervention and postintervention values of the primary and secondary evaluation indices. Repeated measures analyses of variance were used to analyze the changes in elbow ROM, the MEPS, and the SF-36 score at each time point (baseline and at 1, 6, and 12 weeks after the operation). The outcomes were reported as mean (SD) for continuous variables and percentages for categorical variables. Nonnormally distributed variables were depicted using median (25th percentile to 75th percentile). A 2-tailed *P* value <.05 was considered to indicate statistical significance.

## Results

### Recruitment and Research Processes

**Figure 1.**
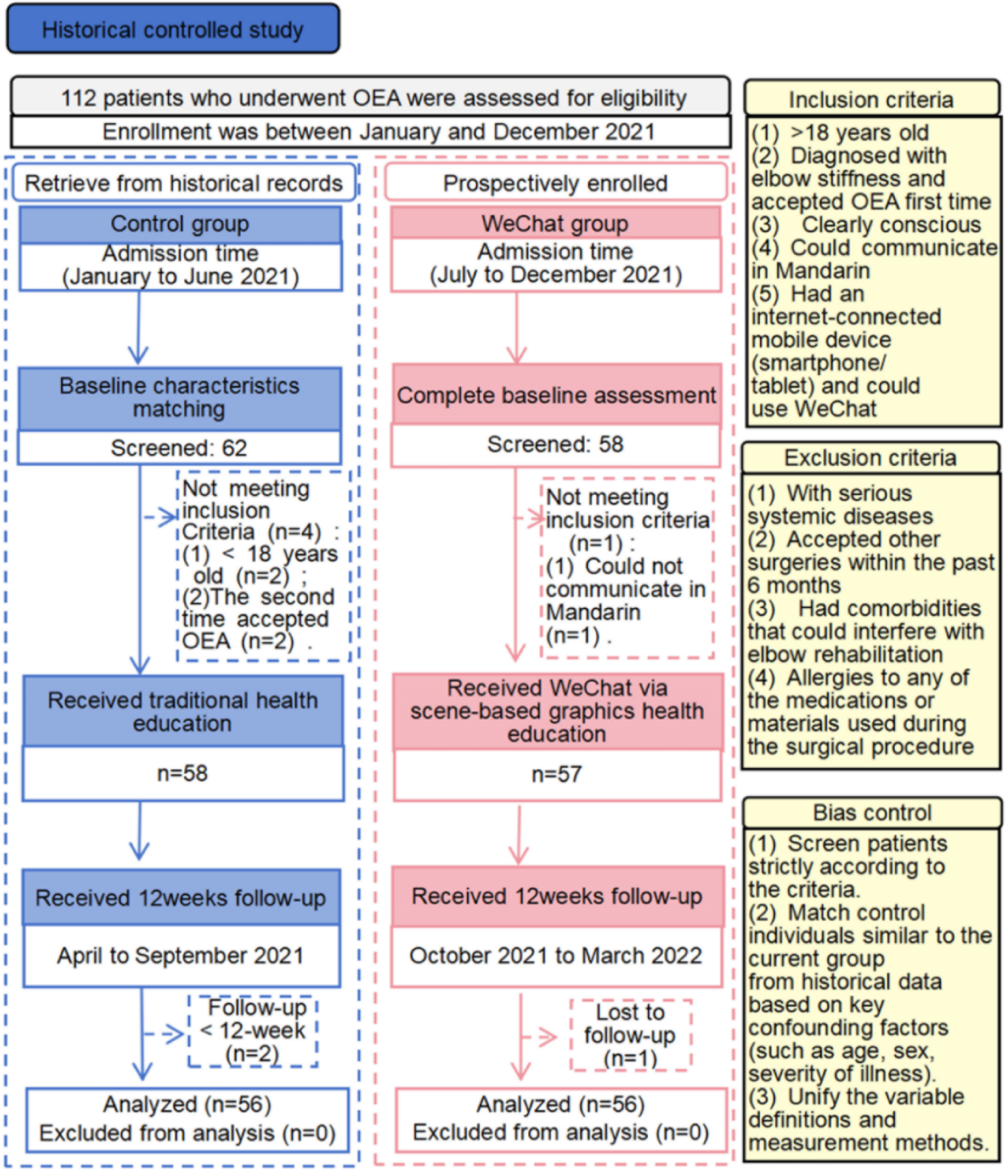
Study flow diagram. OEA: open elbow arthrolysis.

Patients with OEA were recruited in January 2021, and the follow-up process ended in March. Figure 1 shows the CONSORT flowchart of this study, detailing recruitment, assessment, and follow-up. A total of 120 patients were assessed, of whom 112 met the inclusion criteria and were enrolled. During the study, 3 patients dropped out, resulting in a dropout rate of 2.7%. Specifically, 2 patients in the control group failed to comply with elbow rehabilitation, and 1 participant in the WeChat group withdrew for personal reasons.

### Baseline Characteristics of the Participants

The 2 groups exhibited no significant differences (age, *P*=.90; sex, *P*=.34; highest level of education, *P=.*32; BMI, *P*=.43; disease side, *P*=.71; duration of disease, *P*=.26; Duration of surgery, *P*=.93) in any baseline characteristics (Table 1). The average age of all participants was 36.0 years. Of the 112 patients, 59 (52.7%) were female and 53 (47.3%) were male. Among the participants, 44 (39.3%) had a junior college education, and 27 (24.1%) had a university degree or higher. The remaining participants included those with senior high school education (26/112, 23.2%), junior high school education (13/112, 11.6%), and primary school education (2/112, 1.8%); 60 (53.6%) patients had left-sided disease, and 52 (46.4%) had right-sided disease. The average duration of disease and surgery was 8.0 months and 124.96 minutes, respectively.

### Primary Outcome

**Figure 2.**
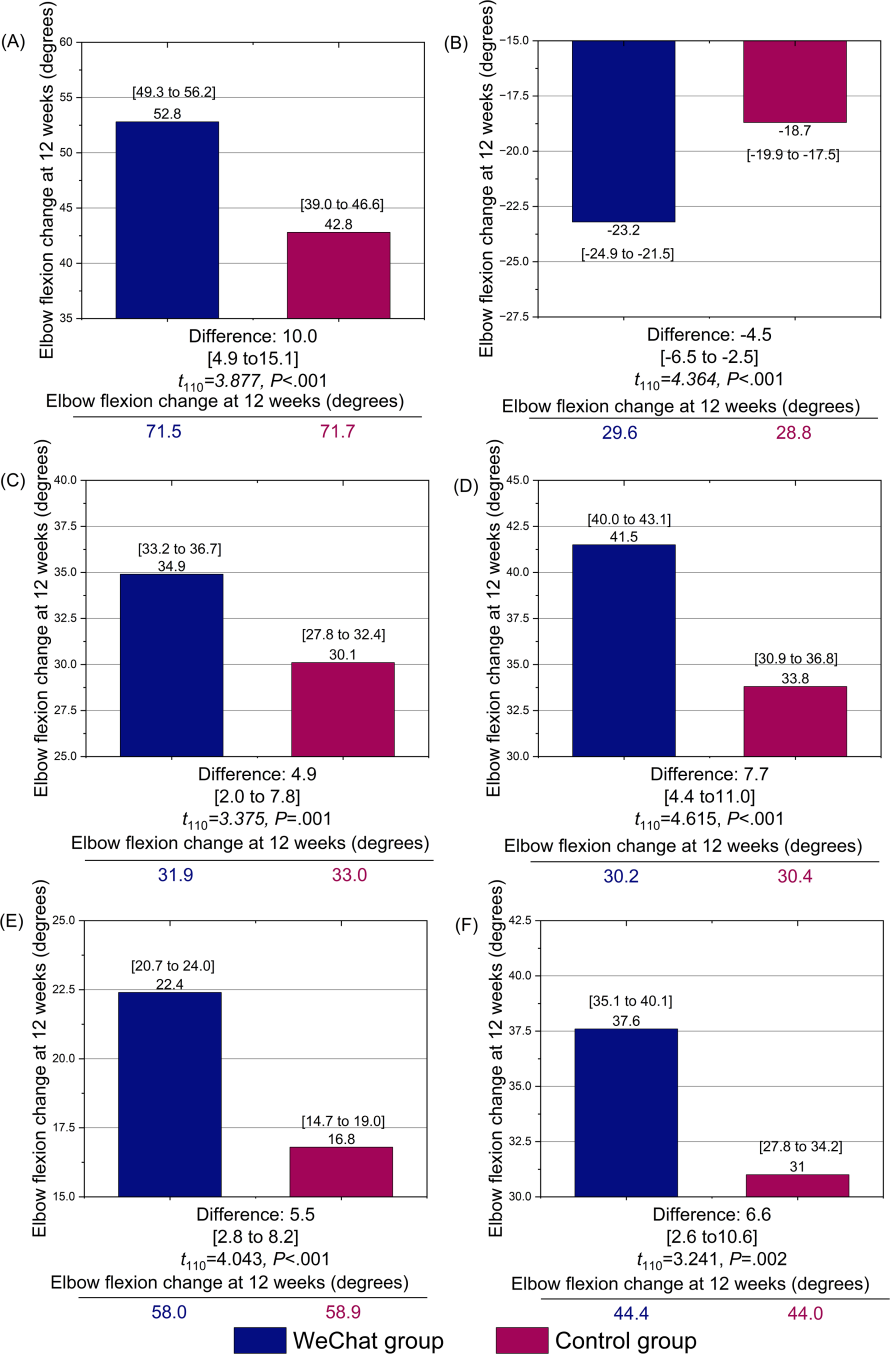
Changes from baseline to 12 weeks postoperatively in elbow and forearm function (flexion/extension, pronation/supination), Mayo Elbow Performance Score, and 36-item Short Form Health Survey scores in the WeChat intervention group (n=56) and control group (n=56), among 112 adult patients with posttraumatic elbow stiffness who underwent open elbow arthrolysis. This historical control study was conducted at a tertiary first-class hospital in Shanghai, China, between January 2021 and March 2022.

**Figure 3.**
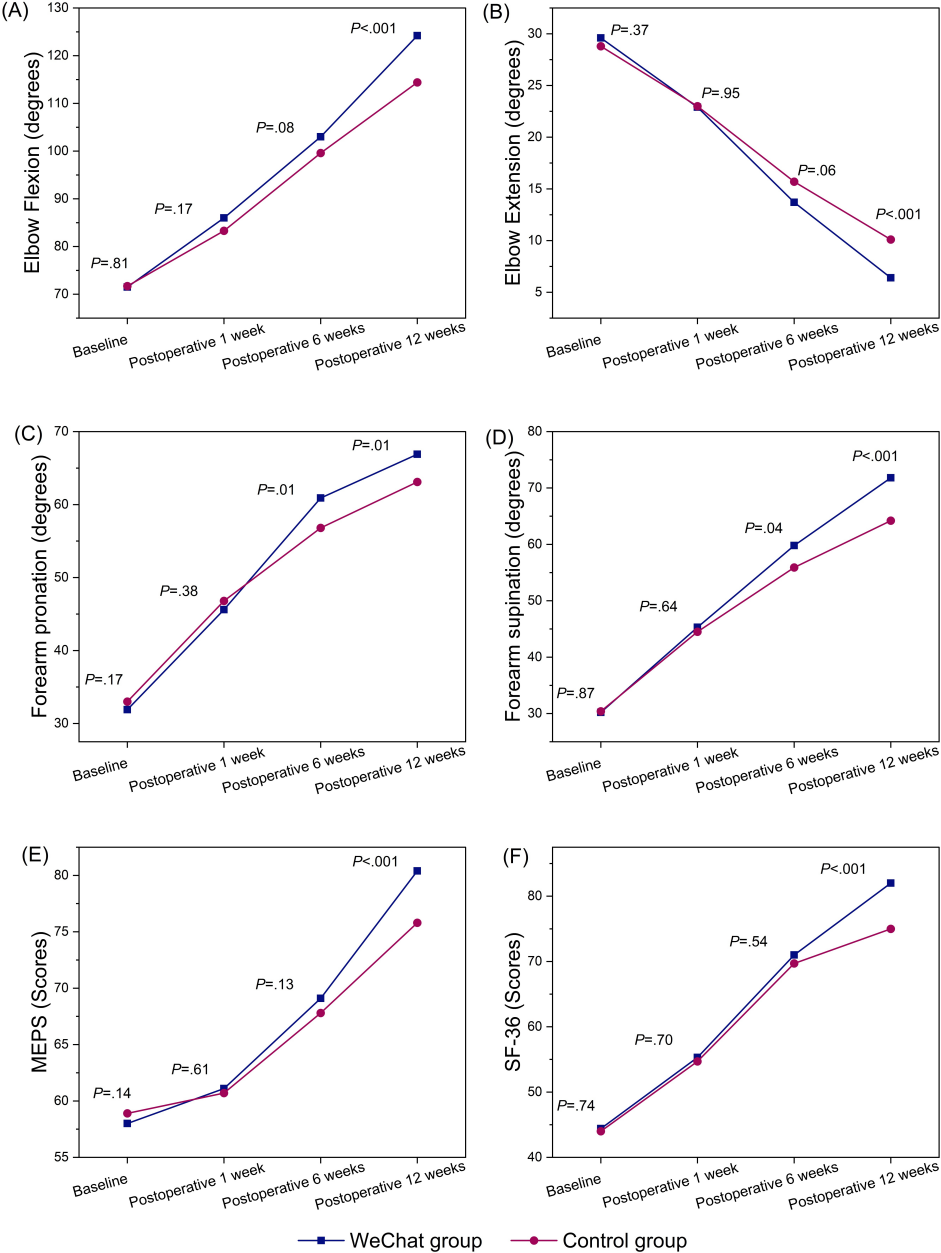
Time-course comparison of elbow and forearm functional outcomes (flexion/extension, pronation/supination), Mayo Elbow Performance Score (MEPS), and 36-item Short Form Health Survey (SF-36) scores between the WeChat group (n=56) and the control group (n=56) among 112 adult patients with posttraumatic elbow stiffness who underwent open elbow arthrolysis. This historical control study was conducted at a tertiary first-class hospital in Shanghai, China, from January 2021 to March 2022.

The study revealed no significant difference in the ROM of the elbow between the 2 groups at the first week after surgery (elbow flexion, *P*=.17; elbow extension, *P*=.95; forearm pronation, *P*=.38; forearm supination, *P*=.64). However, at 6 weeks, the WeChat group demonstrated better forearm pronation (*P*=.01) and forearm supination (*P*=.04) than the control group. At 12 weeks after surgery, compared with the control group, the WeChat group had better ROM in elbow flexion (*P*<.001), elbow extension (*P*<.001), forearm supination (*P*<.001), and forearm pronation (*P*=.006; Table 2). The study also revealed statistically significant differences in terms of time (elbow flexion, elbow extension, forearm pronation, and forearm supination; all *P*<.001), between-group effects (elbow flexion, *P*<.001; elbow extension: *P*=.04, forearm pronation: *P*=.049, forearm supination: *P*<.001), and interaction effects (elbow flexion: *P*=.004, elbow extension: *P*=.005, forearm pronation: *P*=.002, forearm supination: *P*=.003) between the 2 groups, as shown in Table 3. Figure 2 illustrates the difference in elbow ROM between the 2 groups. Figure 3 illustrates the change in elbow ROM over time. Table 4 presents the data on adverse events noted in the groups.

**Table 3.** Trends in elbow and forearm function (flexion/extension and pronation/supination), MEPS^a^, and SF-36^b^ scores in the WeChat intervention and control groups. Outcomes were assessed preoperatively and at 1, 6, and 12 weeks postoperatively in 112 adult patients with posttraumatic elbow stiffness who underwent open elbow arthrolysis. This historical control study was conducted at a tertiary first-class hospital in Shanghai, China, from January 2021 to March 2022, to evaluate the impact of a WeChat-based intervention.

Item	*F*_time_ test (*df*)	*P* value	*F*_between groups _test (*df*)	*P* value	*F*_time×group_ test (*df*)	*P* value
Elbow flexion^c^	493.590 (2.48, 273.22)	<.001	17.718 (1, 110)	<.001	5.083 (2.48, 273.22)	.004
Elbow extension^c^	388.707 (2.63, 289.13)	<.001	4.376 (1, 110)	.04	4.657 (2.63, 289.13)	.005
Forearm pronation^c^	479.929 (2.69, 295.43)	<.001	3.952 (1, 110)	.049	5.200 (2.69. 295.43)	.002
Forearm supination^c^	467.085 (2.53, 277.75)	<.001	17.936 (1, 110)	<.001	5.343 (2.53, 277.75)	.003
MEPS	484.727 (2.00, 220.24)	<.001	4.906 (1, 110)	.03	8.545 (2.00, 220.24)	<.001
SF-36	483.478 (2.29, 251.49)	<.001	4.071 (1, 110)	.046	5.067 (2.29, 251.49)	.005

a MEPS: Mayo Elbow Performance Score.

b SF-36: 36-item Short Form Health Survey.

c Range of motion of the elbow.

**Table 4.** Adverse events in the WeChat group and control group among 112 adult patients with posttraumatic elbow stiffness who underwent open elbow arthrolysis. This historical control study was conducted at a tertiary first-class hospital in Shanghai, China, from January 2021 to March 2022.

Adverse event	WeChat group (n=56), n (%)	Control group (n=56), n (%)
Related to the surgical site	N/A^a^	N/A
Severe bleeding or hematoma	N/A	N/A
Aggravation of wound infection	N/A	N/A
Structural instability	N/A	N/A
Overall physical condition of the patient	N/A	N/A
Serious systemic adverse reactions	N/A	N/A
Serious cardiovascular problems	1 (2)	N/A
Extremely weak or tired	N/A	1 (2)
Abnormal rehabilitation progress	N/A	N/A
Severely limited joint movement and intensified pain	N/A	1 (2)

a N/A: not applicable.

### Secondary Outcomes

This study revealed no significant differences between the 2 groups in MEPS (*P*=.61) or SF-36 (*P*=.69) scores at the first week after surgery. However, the WeChat group had higher MEPS and SF-36 scores at 12 weeks than the control group (all *P*<.001; Table 2). The study also showed statistically significant differences in the time effect (MEPS: *P*<.001 and SF-36 score: *P*<.001), between-group effect (MEPS: *P*=.03 and SF-36 score: *P*=.046), and interaction effect (MEPS: *P*<.001 and SF-36 score: *P*=.005) between the 2 groups, as shown in Table 3. Figure 2 illustrates the differences between the 2 groups in MEPS and SF-36 scores, and Figure 3 shows the changes in MEPS and SF-36 scores over time.

There was no significant difference in the incidence of postoperative complications between the 2 groups (heterotopic ossification: *P*=.80; new onset or exacerbation of ulnar nerve symptoms: *P*>.99; repeat elbow stiffness: *P*=.56). Among the 56 patients in the WeChat group, 2 (4%) developed heterotopic ossification, specifically grade 2A and grade 2C, respectively. In the control group, which also consisted of 56 patients, 3 (5%) had heterotopic ossification, namely, grade 2A, grade 2B, and grade 2C, respectively. The absolute and relative effect sizes were 0% for grade 2A and grade 2B, and 1.8% and 100% for grade 2C, respectively. In both groups, 1 (2%) patient experienced a new onset or exacerbation of ulnar nerve symptoms, with absolute and relative effect sizes of 0%.

Concerning repeated elbow stiffness, 1 (2%) patient was in the WeChat group, and 2 (4%) patients were in the control group. The absolute and relative effect sizes were 1.8% and 50%, respectively.

## Discussion

### Principal Findings

This historical control study investigated the efficacy of integrating WeChat with scene-based graphic health education. The results indicated that the WeChat group demonstrated superior elbow mobility (elbow flexion, extension, forearm pronation, and supination) and functional outcomes (MEPS scores and SF-36 scores) compared with the control group, with treatment effects becoming more pronounced over the postoperative period, particularly at 12 weeks postoperation. These findings suggest that this novel approach can facilitate better and more sustained recovery for patients with posttraumatic elbow stiffness. The method likely helps patients comprehend the rehabilitation process and adhere to the treatment plan, resulting in long-term improvements in elbow function and overall health-related quality of life.

The efficacy of this approach is attributed to immersive comics that reduce cognitive load through a 4-stage information chunking process, and interactive games, which foster adherence to daily tasks. Additionally, WeChat’s real-time feedback minimizes exercise errors, while peer support enhances social support by fostering emotional camaraderie and knowledge sharing among patients. These findings underscore the dual benefits of the approach in improving knowledge retention and sustaining behavioral compliance. It has been shown to be more effective than single-modality interventions.

### Comparison With Prior Work

Interest is often considered the best teacher in the pursuit of learning, and learning serves as a bridge linking us to knowledge and compliance [43]. The form in which messages are delivered significantly influences knowledge absorption [44]. Engaging formats enhance interest and capture readers’ attention, thereby improving learning efficacy [45]. Past research has used various strategies to increase readers’ attention, such as celebrity endorsements, humor, videos, and emoticons [44-46]. Moreover, an article’s format, content, length, and communication style significantly affect readers’ attention [47]. The use of caricatures has been shown to be a key feature in attracting the greatest reader engagement, and posts with images tend to have higher rates of liking and sharing [43]. This study developed a comic book. As previously described, the elbow ROM and MEPS and SF-36 scores in the WeChat group were significantly better than those in the control group. Its effectiveness is rooted in cognitive load theory, which posits that the human brain has a limited information-processing capacity at any given time, categorized into intrinsic cognitive load (task/person-specific information complexity), extraneous cognitive load (suboptimal presentation conditions), and germane cognitive load (working memory resources allocated to process intrinsic load) [48]. The comic book’s advantages align closely with this framework. First, the comic systematically partitions complex rehabilitation protocols into 4 graduated modules, reflecting Sweller’s simple-to-complex instructional paradigm [48]. This phased decomposition mitigates intrinsic cognitive load by translating daunting tasks (eg, 300 groups of motions that need to be mastered in the early postoperative stage) into sequentially manageable subtasks. For example, stepwise motion schematics visually deconstruct exercise routines, reducing the cognitive effort required to interpret textual instructions. It also significantly reduces patients’ psychological aversion to excessive task volume, thereby alleviating intrinsic cognitive load [47]. Second, comic illustrations serve as a perceptual bridge for abstract medical concepts. Dynamic visuals and narrative threads enhance retention by fostering immersive learning environments [49-51] consistent with Mayer’s signaling principle [48,52]. Color-coded anatomical annotations and symbolic icons (eg, distinct shades denoting elbow components) adhere to the redundancy principle [48], ensuring visual-textual synergy while avoiding information duplication. Visual cues, such as arrows highlighting hinged external fixator adjustments, further optimize information hierarchy, minimizing extraneous load associated with identifying critical details [49]. These design elements enhance aesthetic appeal and function as memory anchors, corroborated by research on visual mnemonic efficacy [49]. Third, the comic’s narrative structure cultivates patient engagement through relatable, character-driven scenarios, contrasting with the abstract nature of conventional educational materials [50,51,53]. By embedding rehabilitation guidance within plausible story arcs, such as a protagonist’s recovery, the comic amplifies germane load through personal relevance, a mechanism supported by narrative transportation theory [53]. This emotional resonance deepens information encoding and may improve treatment adherence by transforming instructional content into personally meaningful narratives [48].

High participation is a fundamental prerequisite for effective and rapid knowledge acquisition [54]. Self-determination theory, based on 3 innate and universal psychological needs, competence, autonomy, and relatedness, explains this phenomenon [55].

When individuals’ psychological needs are met, they engage in learning more actively [55]. Optimizing the learning environment helps satisfy these needs [55]. Gamification can fulfill these needs by providing an engaging environment that effectively drives participants’ motivation [52]. Elements such as goal-setting, recording or affirmation of performance, and rewards for achievement influence learning efficacy [55]. In this study, interactive games were developed for patients with OEA. While elbow ROM, MEPSs, and SF-36 scores showed no significant intergroup differences 1 week postoperation, the gamified design in the WeChat group effectively addressed patients’ early psychological needs, establishing a solid foundation for long-term therapeutic benefits. Specifically, games such as the “Picture-Based Cloze Test” were implemented to reinforce critical rehabilitation concepts. Through repeated practice sessions, patients engaged with challenging content until mastery was achieved, at which point a congratulatory message (“Well Done!") would appear on the PowerPoint screen. This iterative approach, designed to balance difficulty with achievable goals, aimed to foster intrinsic motivation by leveraging the psychological need for competence—the satisfaction of which, as posited by self-determination theory, drives self-determined behavior [55]. By embedding enjoyable elements into knowledge acquisition, the games promoted deeper engagement with rehabilitation materials, consistent with the self-determination theory’s emphasis on intrinsic motivation as a primary driver of sustained learning [55]. Crucially, the design prioritized autonomy support by allowing patients to progress at their own pace, thereby satisfying the theoretical need for autonomy. Additionally, as patients received encouragement and praise from the interactive games, autonomy-supportive extrinsic motivation further enhanced their sense of autonomy, leading to a deeper internalization of the rehabilitation process and making it more meaningful and self-driven [55]. Finally, peer-assisted interactions (eg, mutual encouragement) within the games satisfied the need for relatedness, improving both intrinsic and extrinsic motivation for long-term adherence to rehabilitation protocols [55]. The interactive nature of the games allows patients to receive immediate feedback, which is crucial for learning and retaining rehabilitation exercises. This feedback loop reinforces correct techniques and provides a sense of accomplishment, further motivating patients to continue their rehabilitation efforts. By addressing these psychological needs, interactive games make rehabilitation more enjoyable and effective. This multifaceted approach contrasts starkly with traditional health education, which often falters due to its one-size-fits-all delivery. Postoperative patients, already stressed by surgery, may resist static written materials or passive video tutorials that offer no opportunity for agency [45]. Moreover, traditional methods rely heavily on nursing staff’s communication skills, introducing variability in information retention [27]. The absence of immediate feedback and adaptive pathways further limits patients’ ability to build competence or sustain engagement.

Behavioral oversight plays a crucial role in health management, is equally pivotal in elbow rehabilitation, and is firmly grounded in social support theory [56]. This theory poses that social support involves exchanging resources between individuals to improve the recipient’s health, functioning, and well-being [57]. It can be divided into instrumental, emotional, informative, and other forms of support. When individuals are supported by their social environment, such as health care professionals, family members, or even peers, their behavior can be strengthened [57]. In rehabilitation, behavioral oversight ensures that patients follow treatment plans, provides encouragement, and identifies challenges for timely adjustments. Such oversight fosters a structured, supportive environment that enhances adherence and recovery. Recently, WeChat has emerged as a pivotal tool in health education and disease management, garnering widespread acceptance [19-23,25-32]. Enhancing health information dissemination and assimilation through WeChat has become a focal point worthy of further research [43,44]. This study highlights key aspects of behavioral oversight via WeChat. At 6 and 12 weeks postoperatively, the WeChat group showed significantly better elbow ROM, MEPS, and SF-36 scores, along with a lower complication rate. This can be explained as follows: First, WeChat creates a structured professional support system that provides instrumental patient support [57]. It enables ongoing communication between researchers and patients after discharge, facilitating real-time monitoring and feedback, thereby ensuring seamless educational support. Procedures such as “daily check-in reminders“ prompt patients to maintain their rehabilitation routines. By setting daily goals and incorporating knowledge consolidation, patients are more likely to adhere to their rehabilitation plans and achieve better outcomes. The “Error Correction” and “Daily Q&A” features serve as educational tools and behavioral assessments. Researchers can use the results to identify areas where patients may struggle and provide targeted support and guidance. This closed loop of “monitoring-feedback-intervention” aligns with the theory’s emphasis on organized support to enhance self-regulation, ensuring patients receive continuous, tailored advice to maintain adherence [57]. Second, WeChat’s social features foster peer emotional support networks, a core component of emotional support in the theory [57]. Patients who share rehabilitation videos or group pain management experiences engage in reciprocal emotional validation. For example, a patient’s demonstration of achieving 100° elbow flexion at 8 weeks postoperation often triggers observational learning and mimicry among peers, enhancing collective self-efficacy through vicarious experience and motivating sustained adherence to treatment plans. Third, WeChat’s data-tracking capabilities enable dynamic support strategies rooted in informational support [57]. Using WeChat for behavioral oversight also provides valuable data for researchers to analyze patient behavior patterns and treatment outcomes, yielding insights that can further refine rehabilitation strategies and improve patient care.

### Strengths and Limitations

This study has several strengths. First, it introduces a novel and innovative health education approach to elbow rehabilitation by incorporating comics, integrative games, and WeChat. The approach enhances patient engagement and comprehension, ensuring better retention and understanding of rehabilitation procedures. Second, the study emphasizes the importance of behavioral oversight in health management, which is often overlooked in traditional health care settings. By leveraging the capabilities of the WeChat platform, it provides a feasible solution for continuous, personalized monitoring of patient behavior postdischarge.

Importantly, this study has certain limitations. First, the patient follow-up period was limited to 12 weeks, which does not provide insight into long-term outcomes. Although this relatively short time frame restricts our understanding of sustained changes, the data collected during these 12 weeks can offer valuable early indications. Several considerations can help mitigate the potential impact of this limitation on the interpretation of our results. First, although the short follow-up restricts our understanding of long-term changes, the data collected during these 12 weeks can provide valuable early indications. These findings can inform future investigations with extended follow-up periods to validate and extend upon our findings. Additionally, despite the absence of long-term data, the observed short-term effects remain useful for guiding clinical practice. Clinicians can use these early outcomes to make informed decisions regarding patient care while remaining attentive to potential differences in long-term results. Second, the study was conducted at a single center, which may limit the generalizability of the findings. The unique characteristics of the patient population and the health care system at this center could influence the results and their applicability to other settings. Another limitation is the potential for self-reporting bias in the behavioral monitoring component. Patients may not always accurately report their behavior, which could affect the reliability of the data.

### Future Directions

For future research, it would be beneficial to extend the follow-up period to assess the long-term efficacy of the rehabilitation program. This would provide a more comprehensive understanding of the sustained impact of the intervention on patient outcomes. Additionally, future studies should replicate this study across multiple centers with diverse patient demographics and health care systems. Such efforts would help determine whether the observed benefits are consistent across different environments and populations. Also, this study’s approach could be applied to other types of rehabilitation or different patient populations to assess its generalizability. Furthermore, to minimize self-reporting bias, future studies could incorporate objective measures, such as wearable devices or other technologies, to track patient activity levels and adherence to rehabilitation protocols more accurately. Finally, investigating the cost-effectiveness of using comics, integrated games, and WeChat in health education would provide valuable insights for health care providers considering the implementation of similar programs.

### Conclusion

This study demonstrates that WeChat-based plus scene-graphical health education effectively communicates information to patients who have undergone OEA. This approach has been shown to enhance elbow ROM, functionality, and overall quality of life. Furthermore, it offers a platform for patients to address challenges and support one another.

## Supplementary material

10.2196/58218 Multimedia Appendix 1 Protocol summary and additional information.

10.2196/58218 Multimedia Appendix 2 Comic materials for the study groups.

10.2196/58218 Checklist 1 CONSORT (Consolidated Standards of Reporting Trials) checklist.

## Abbreviations

CONSORT: Consolidated Standards of Reporting Trials
HbA_1c_: glycated hemoglobin
MEPS: Mayo Elbow Performance Score
OEA: open elbow arthrolysis
ROM: range of motion
SF-36: 36-item Short Form Health Survey
