# Supplementary material for: Effectiveness of WeChat-Based Plus Scene-Graphics Health Education for Rehabilitation After Open Elbow Arthrolysis: Historical Control Study
Source: JMIR Form Res. 2025 Oct 21;9:e58218. doi: 10.2196/58218 (PMC12605337; doi:10.2196/58218)
Supplement: Multimedia Appendix 1 [file formative-v9-e58218-s001.docx]

Effectiveness of WeChat-based plus scene-graphics health education for rehabilitation after open elbow arthrolysis: Protocol for a historical control study

Title: Effectiveness of WeChat-based plus scene-graphics health education for rehabilitation after open elbow arthrolysis: A historical control study

Brief Title: Postoperative Elbow rehabilitation

**Correspondence:**

**List of Abbreviations**

| CONSORT | Consolidated Standards of Reporting Trials |
| --- | --- |
| OEA | Open elbow arthrolysis |
| MEPS | Mayo Elbow Performance Score |
| SF-36 | Short Form 36-item Health Survey |

**Protocol Summary**

| **Short Title** | Postoperative Elbow rehabilitation |
| --- | --- |
| **Objectives** | This study aims to assess the efficacy of WeChat–based plus scene–graphics health education following open elbow arthrolysis. |
| **Study Design** | This study is a single-center, interventional, historical control study. |
| **Ethical Registration Number** | 2020–028 |
| **Clinical Research Registration Number** | ChiCTR2000036004 |
| **Study Center** | Shanghai sixth people’s hospital, China Shanghai |
| **Primary Outcome** | The primary outcome was the difference in elbow range of motion after 1-week, 6-weeks and 12-weeks post-operative. |
| **Secondary Outcome** | 1-week, 6-weeks and 12-weeks of Psysiological function in quality-of-life scores(SF-36), elbow function by using Mayo Elbow Performance Score(MEPS), assessing the incidence of complications through clinic manifestations and examination results. |
| **Population** | 112 patients diagnosed as elbow stiffness that need to undergo open elbow arthrolysis will be recruited and enrolled into the WeChat group (n=56) or the Control group (n=56). |
| **Study Duration** | 15 months |
| **Follow-up visit** | 1-week, 6-weeks and 12-weeks post-operative |
| **Intervention Group** | Received health education using WeChat and scene–graphics. The information in 4-part comics was shared via a WeChat public account. The patients got information from the account and had daily lessons during hospitalization, and online instruction in a WeChat group after discharge until 12 weeks post-operation. |
| **Control Group** | traditional health education  Received the usual health education provided by the hospital, which includes verbal instructions and electronic materials. Patients in this group did not receive any WeChat-based or scene-graphics education. |
| **Main Inclusion Criteria** | 1. Patients must be aged >18 years old.  2. Must have a diagnosis of elbow stiffness requiring surgical intervention.  3. Must be willing and able to provide informed consent.  4. Must be able to understand and communicate in Mandarin, as the WeChat public account and group will be conducted in these languages.  5. Must have access to a smartphone with WeChat application installed for the intervention group participants. |
| **Main Exclusion Criteria** | 1. Patients with a history of psychiatric disorders or cognitive impairments that would interfere with the ability to comply with the study protocol.  2. Patients who have undergone previous elbow surgeries or have other medical conditions that could affect elbow function.  3. Pregnant women or those planning to become pregnant during the study period.  4. Individuals with known allergies to any of the medications or materials used during the surgical procedure.  5. Patients who are participating in other clinical trials simultaneously or have done so within the past 6 months. |
| **Allocation** | Eligible patients were split into two groups by admission time: the control group (56 patients enrolled from Jan.–Jun. 2021) and the WeChat group (56 patients enrolled from Jul.–Dec. 2021). |
| **Statistical Methodology** | The statistical analysis was conducted using SPSS software version 26.0. The primary outcome measures were elbow range of motion (ROM), which were assessed at baseline, at discharge, and at 1, 6, and 12 weeks post-operation. Secondary outcomes included quality of life, elbow function, and postoperative complications. The data was analyzed using descriptive statistics to summarize the demographic and clinical characteristics of the participants. Inferential statistics, including t-tests for continuous variables and chi-square tests for categorical variables, were used to compare the differences between the control and WeChat groups. Repeated measures analysis of variance (ANOVA) was conducted to evaluate the changes in ROM over time within each group and between the groups. The significance level was set at p<0.05 for all statistical tests. |
| **Safety** | Adverse events will be summarized for each group. The safety analysis will focus on the frequency and severity of any complications that arise during the study period. This includes, but is not limited to, infections, allergic reactions, and any other unexpected medical events that may occur. The safety data will be analyzed to determine if there is a significant difference in the incidence of adverse events between the control and WeChat groups. The results will be reported in accordance with the CONSORT guidelines for randomized controlled trials, ensuring transparency and completeness of the safety data. |
| **Result** | Elbow flexion angle: WeChat group 71.5 (SD 4.2) to 124.2 (SD 11.7) °; Control group 71.7 (SD 4.6) to 114.4 (SD 13.6) ° (diff 10 °, 95% CI 4.9–15.1, P <.001). Elbow extension angle : WeChat group 29.6 (SD 6.0) to 6.4 (SD 2.5) °; Control group 28.8 (SD 3.8) to 10.1 (SD 3.4) ° (diff -4.5 °, 95% CI -6.5– -2.5, P <.001). Forearm pronation angle: WeChat group 31.9 (SD 4.0) to 66.9 (SD 7.3) °; Control group 33.0 (SD 4.2) to 63.1 (SD 7.2) ° (diff 4.9 °, 95% CI 2.0 –7.8, P =.001). Forearm supination angle: WeChat group 30.2 (SD 3.7) to 71.8 (SD 4.8) °; Control group 30.4 (SD 4.1) to 64.2 (SD 9.8) ° (diff 7.7 °, 95% CI 4.4–11.0, P <.001). MEPI score: WeChat group 58.0 (SD 3.7) to 80.4 (SD 5.7); Control group 58.9 (SD 2.8) to 75.8 (SD 6.9) (diff 5.5 points, 95% CI 2.8–8.2, P <.001). SF - 36 score: WeChat group 44.4 (SD 6.6) to 82.0 (SD 7.1); Control group 44.0 (SD 6.4) to 75.0 (SD 11.2) (diff 6.6 points, 95% CI 2.6–10.6, P =.002). |

Content

[List of Abbreviations 1](#_Toc5648)

[Protocol Summary 1](#_Toc8973)

[1. Introduction and Rationale 5](#_Toc6776)

[1.1 Introduction 5](#_Toc12802)

[1.2 Rationale 8](#_Toc10032)

[2. Study Design and Purpose 10](#_Toc13487)

[2.1 Study Design 10](#_Toc9164)

[2.2 Study Purpose 10](#_Toc13211)

[3. Study Population 11](#_Toc16618)

[3.1 Enrollment Population 11](#_Toc9790)

[3.2 Inclusion and exclusion criteria. 11](#_Toc17452)

[3.3 Informed Consent 14](#_Toc24464)

[3.4 Early Withdrawal of subjects 15](#_Toc20744)

[3.5 Replacement of participant 15](#_Toc2086)

[3.6 Early termination of the study 15](#_Toc31974)

[3.7 Interim analyses 16](#_Toc23113)

[4.Study Procedures 16](#_Toc12649)

[4.1Participant recruitment 17](#_Toc14242)

[4.2 Blinding 17](#_Toc13786)

[4.3 Intervention 17](#_Toc11637)

[4.4 Study Duration 25](#_Toc16652)

[5.Data 29](#_Toc25472)

[5.1 Data Collection 29](#_Toc16174)

[5.2 Data Transmission 29](#_Toc4851)

[5.4 Data Storage 30](#_Toc4970)

[5.5 Data Sharing 30](#_Toc20005)

[5.6 Cancellation of Participation 30](#_Toc6979)

[5.7 Researcher Confidentiality 31](#_Toc26350)

[5.8 Data Retention Period 31](#_Toc10761)

[6. Safety 31](#_Toc28880)

[6.1 Related to the surgical site 31](#_Toc9447)

[6.3Abnormal rehabilitation progress 33](#_Toc1357)

[6.2 Safety of Data Collection 34](#_Toc27726)

[7. Study Monitoring 36](#_Toc25635)

[7.2 Collect patient's feedback 36](#_Toc26060)

[8. Ethical Considerations 36](#_Toc5706)

[Appendix 1. 38](#_Toc20883)

[Informed Consent 38](#_Toc12767)

[Introduction 39](#_Toc12600)

[Study Procedure 43](#_Toc3680)

[Benefits of participating in the study 44](#_Toc29049)

[Research-related updates 45](#_Toc29679)

[Cost for participate this study 45](#_Toc22271)

[Confidentiality and privacy 46](#_Toc25963)

[Right and responsibility 46](#_Toc28287)

[1)Right : 46](#_Toc25768)

[2)Responsibility : 46](#_Toc687)

[Attention 46](#_Toc18699)

[Related to the surgical site 47](#_Toc28372)

[Abnormal rehabilitation progress 48](#_Toc17487)

Appendix2. 50

Appendix 3.................................................................................................................................... ..54

[References 55](#_Toc6922)

1. **Introduction and Rationale**

**1.1 Introduction**

Normal elbow mobility serves as an essential and fundamental condition in our daily life. It is the cornerstone that enables us to perform a wide array of activities with ease. Elbow stiffness, unfortunately, is a rather prevalent issue, and its incidence is surprisingly high. In fact, it has been found to affect as many as 56% of individuals subsequent to elbow trauma [1, 2]. This condition is marked by a significant limitation in the range of motion or rotation of the elbow joint, which in turn results in the inability of the elbow to fulfill the patient's diverse requirements for daily activities, including but not limited to simple tasks like dressing and eating, as well as more complex ones related to work and various forms of entertainment [3, 4].

Open elbow arthrolysis (OEA) has emerged as the most commonly employed surgical intervention in such cases. Extensive research and clinical practice have demonstrated its remarkable efficacy in restoring the elbow's function and range of motion [3, 4]. During the surgical procedure, surgeons can achieve the release of the elbow by carefully removing bony impediments or scar tissue within the olecranon fossa. This meticulous process aims to adjust the flexion of the elbow to more than 130° and the extension to less than 10°, thereby optimizing the joint's functionality [5, 6].

However, it is of utmost importance to note that postoperative rehabilitation plays an indispensable role in the overall process of elbow function recovery [4 - 8]. A substantial and ever - growing body of evidence clearly indicates that when postoperative rehabilitation is carried out in a timely and appropriate manner, it paves the way for highly favorable outcomes. On the contrary, if the rehabilitation process is inadequate, it can lead to a host of severe problems related to elbow recovery [4 - 8]. These problems are not limited to just a minor setback but can involve various postoperative complications that can significantly impact the patient's quality of life. Such complications include heterotopic ossification (HO), which can cause additional pain and restricted movement. Additionally, new onset or exacerbation of ulnar nerve symptoms can occur, leading to nerve - related issues such as numbness or weakness in the arm. Moreover, there is a significant risk of repeat elbow stiffness, which can be a major setback for the patient's recovery journey [3, 9 - 11].

According to incomplete yet telling statistics, the situation is quite concerning. The incidence of postoperative elbow issues stands at a staggering 56 - 87% [5]. The probability of renewed elbow stiffness is also alarmingly high, ranging from 8.4 - 47% [5, 6]. Furthermore, the rate of secondary surgery, which is often required to address these complications, is between 10 - 34% [6, 7]. To make matters worse, the rate of patient dissatisfaction due to these issues reaches 21% [7]. These figures clearly emphasize the need to enhance the precision of postoperative rehabilitation and ensure better patient adherence to the rehabilitation protocols.Nevertheless, achieving perfect postoperative rehabilitation is an extremely challenging task. This is primarily because the process is not only prolonged but also highly intricate [6, 12, 13]. Standard postoperative rehabilitation typically spans a period of one year and is systematically divided into three distinct phases [6, 14, 15]. The first phase, known as the acute phase, commences on the very first day after the surgery and extends up to 6 weeks. During this initial and crucial period, the rehabilitation process involves a series of passive, assisted, and active elbow flexion and extension motions [6, 14, 15]. To be more specific, on the first day, patients are recommended to start with 30 such movements. As the days progress, this number is increased by 30 each day until a total of 300 movements are achieved on a daily basis [6, 14]. The second phase, which lasts from 6 weeks to 3 months after the surgery, is designated as the subacute phase. During this period, the daily requirement for these elbow movements remains at 300, ensuring a consistent and continuous rehabilitation effort [14].The third and final phase, the functional phase, lasts from 3 months up to one year after the operation. This phase is particularly significant as it involves the addition of weight - bearing practices to the rehabilitation routine. Moreover, the intensity of the rehabilitation activities is gradually increased on a daily basis during this period [14, 15].It is important to recognize that in addition to the initial rehabilitation process that takes place within the hospital environment, patients are also required to continue their rehabilitation efforts at home. This home - based rehabilitation is an integral part of the overall recovery process. As a result, having a comprehensive and highly accurate grasp of the relevant knowledge related to rehabilitation is absolutely crucial for ensuring effective rehabilitation outcomes [13, 14, 16].

However, in recent times, there has been a significant increase in the prevalence of Enhanced Recovery After Surgery (ERAS) protocols [17 - 18]. While these protocols have brought about several benefits, one of the consequences has been a reduction in the duration of hospital stays. This reduction, unfortunately, has created a new challenge as traditional health education methods have proven to be insufficient for patients to gain the adequate amount of knowledge required for successful rehabilitation. Therefore, there is an urgent need to develop more efficient and feasible health education approaches to address this critical issue.

**1.2 Rationale**

The rationale behind the development of these new approaches is to ensure that patients are well-equipped with the necessary knowledge and skills to continue their rehabilitation process effectively at home. This is particularly important given the shortened hospital stays and the critical role that self-management plays in the overall recovery. The new health education methods must be designed to be easily accessible, engaging, and tailored to the individual patient's needs and learning pace. They should also incorporate feedback mechanisms to monitor patient progress and adapt the educational content accordingly. By addressing these needs, it is hoped that patients will be able to achieve better rehabilitation outcomes and a higher quality of life post-surgery.

In recent years, telemedicine services have witnessed a growing popularity in the realm of health education. Healthcare professionals leverage electronic information and communication technology to offer and support healthcare services to patients, without being restricted by the patients' location or transportation issues [19]. WeChat, being one of the most widely and frequently utilized free platforms in China [20 - 22], is capable of providing convenient telemedicine services. It can accommodate various data types including text, voice calls, videos, and images, enabling convenient and clear communication [21], thereby attracting a large number of users. By the end of 2020, there were 1.2 billion monthly active users globally [22, 23], and these users are more vigorously seeking online healthcare and health information exchange [24]. A prior survey indicated that one - third of the participants regularly read health - information - related articles on WeChat [25], and 98.53% of them used WeChat to search for health information [24]. This platform has been applied in the health management of numerous conditions such as public health literacy [26], cancer [27, 28], asthma [29], chronic obstructive pulmonary disease [30], hypertension [31], myopia [32], coronary artery disease [33], etc. Nevertheless, no research has concentrated on its application in the postoperative rehabilitation of patients with elbow stiffness.

Medical humanities is an interdisciplinary multifaceted field that combines scientific knowledge and skills with respectful, compassionate care that is sensitive to the values, autonomy, and cultural backgrounds of patients and their families[34]. Medical comics are among the numerous approaches in medical humanities that have the potential to address challenging situations within medical settings [34,35]. Medical comics have been utilized to promote adolescent peer support and healthy sexual behaviors [35], raise awareness about diabetes [36], improve the quality of palliative care [37], and increase whole-body donation awareness[38]. Scene-based graphics are a type of approach that can simplify knowledge points into comics and can deliver messages in an engaging manner through various humorous scenarios so that the knowledge becomes easily understandable and memorable. The graphics have converted activities of daily living scales into comics for rapid and straightforward evaluation [39]. Additionally, interactive games have been added into it which align with the corresponding knowledge [40]. Interactive games can be designed according to the key point of knowledge and the characteristics of the learners, which can encourage participants to engage in learning and apply their knowledge more skillfully. However, this approach is in the initial stage and is rarely employed in postoperative rehabilitation. This study was conducted as a historical control study to evaluate the efficacy of WeChat-based plus scene-graphics health education in the postoperative rehabilitation of open elbow arthrolysis.

**2. Study Design and Purpose**

**2.1 Study Design**

The study was designed as a historical control study to assess the effectiveness of a WeChat-based health education program that incorporates scene-graphics in the rehabilitation process following open elbow arthrolysis.

The study was conducted over a period of 15months, with a total of 112 patients who underwent open elbow arthrolysis being enrolled. The patients were divided into two groups: the WeChat group, which received the WeChat-based plus scene-graphics health education, and the control group, which received traditional educational intervention. The primary outcome measure was the improvement in elbow range of motion (ROM) at the end of the 1-week, 6-weeks, 12-weeks post-operative period. Secondary outcomes included , the quality of life , elbow function and the rate of complications. Data were collected at baseline, at 1-week, 6-weeks, and 12-weeks postoperatively. The study aimed to determine whether the innovative educational approach could enhance patient recovery and engagement in rehabilitation, leading to better clinical outcomes.

**2.2 Study Purpose**

**2.2.1Primary purpose**

The primary objective of this study was to evaluate the effectiveness of a novel WeChat-based health education program that integrates scene-graphics in the rehabilitation process of patients undergoing open elbow arthrolysis. By comparing the outcomes of patients in the WeChat group with those in the control group, the study sought to ascertain if the innovative approach could lead to significant improvements in elbow range of motion, quality of life, and function, as well as a reduction in the rate of complications.

**2.2.2Secondary purpose**

The secondary purpose was to explore the potential of interactive educational tools, such as games, to increase patient engagement and adherence to rehabilitation protocols, thereby potentially accelerating recovery and enhancing overall patient satisfaction.

**3. Study Population**

**3.1 Enrollment Population**

The enrollment population consisted of patients who met the inclusion criteria, which included individuals with elbow stiffness or limited range of motion following elbow trauma or surgery. Exclusion criteria were applied to ensure the safety and validity of the study, such as patients with severe medical conditions that could interfere with rehabilitation or those who were unable to comply with the study protocol. The demographic characteristics of the enrolled patients, including age, gender, and the nature of their elbow conditions, were recorded to ensure a balanced comparison between the WeChat group and the control group. The study's recruitment process was designed to be inclusive yet rigorous, aiming to gather a diverse yet comparable sample for the analysis of the educational intervention's impact on rehabilitation outcomes.

**3.2 Inclusion and exclusion criteria.**

**3.2.1 Inclusion criteria**

(1) older than 18 years old;

(2) had been diagnosed with elbow stiffness;

Diagnostic Criteria for Elbow Joint Stiffness

1)Medical History

Trauma (fractures, dislocations, soft - tissue injuries), surgical history of the elbow, and diseases (rheumatoid arthritis, osteoarthritis, infectious diseases).

2)Symptoms

Limited range of motion (less than normal flexion - extension and pronation - supination), pain during movement, and joint swelling.

4)Physical Examination

Visual inspection for swelling, deformity, or scars; palpation for tenderness, temperature, or nodules; measurement of active and passive range of motion and observation of abnormal sounds or blockages.

5)Imaging Examinations

X - ray for bone structure (joint space, bone spurs, fractures), CT for detailed bone morphology, and MRI for soft - tissue lesions (cartilage damage, synovitis, tendon tears, or adhesions).

(3)had undergone open elbow arthrolysis for the first time;

(4)were clearly conscious(Glasgow Coma Scale score of 15);

(5) could communicate with a researcher in Chinese (Mandarin) normally;

(6) had a mobile device (e.g., smartphone or tablet) with an internet connection and were capable of using WeChat.

**3.2.2 Exclusion criteria**

(1) Patients with serious systemic diseases (e.g., uncontrolled hypertension with a blood pressure consistently above 180/110 mmHg, poorly controlled diabetes with HbA1c > 9%, or active malignancy) ;

Here are some systemic diseases that should be excluded in this study:

1) Cardiovascular System Diseases

Severe Coronary Heart Disease: Such as having had a myocardial infarction recently, or suffering from severe unstable angina where angina pectoris occurs frequently even at rest, and there are multiple severe coronary artery stenoses.

Severe Cardiac Arrhythmia: For example, sustained ventricular tachycardia, a history of ventricular fibrillation, or sick sinus syndrome without a pacemaker installed, resulting in patients often experiencing dizziness, blackouts or even syncope.

Severe Heart Failure: Like patients with New York Heart Association (NYHA) class Ⅲ - Ⅳ heart failure, presenting with obvious dyspnea that occurs even with slight activity or at rest, accompanied by peripheral edema, hepatomegaly and other symptoms of systemic congestion.

2) Respiratory System Diseases

Acute Exacerbation of Severe Chronic Obstructive Pulmonary Disease (COPD): Patients experience severe dyspnea, along with increased respiratory rate, use of accessory muscles for breathing. Blood gas analysis shows severe hypoxemia and/or hypercapnia, such as arterial partial pressure of oxygen (PaO₂) being lower than 50 mmHg and partial pressure of carbon dioxide (PaCO₂) being higher than 50 mmHg.

Status Asthmaticus: Refers to a severe asthma attack that does not relieve after conventional treatments such as bronchodilators, lasting for more than 24 hours. Patients show extreme dyspnea, orthopnea, inability to speak in complete sentences, and widespread wheezing in both lungs can be heard. In severe cases, the wheezing may weaken or disappear due to severe airway obstruction (silent chest).

Acute Respiratory Distress Syndrome (ARDS): Usually induced by severe infections, trauma and other factors. Patients have progressive dyspnea, a significant decrease in the oxygenation index (PaO₂/FiO₂), generally less than 200 mmHg, and chest imaging shows diffuse infiltrates in both lungs.

3) Digestive System Diseases

Acute Severe Pancreatitis: Patients have severe upper abdominal pain that can radiate to the lower back, accompanied by nausea and vomiting. Examinations may reveal a significant increase in serum amylase and lipase, often more than three times the upper limit of normal. Meanwhile, multiple organ dysfunction syndrome (MODS) may occur, such as acute kidney injury, respiratory failure, etc.

Decompensated Cirrhosis of the Liver with Severe Complications: Such as the presence of massive ascites causing abdominal distension, esophageal and gastric variceal bleeding manifested as hematemesis and/or melena, hepatic encephalopathy where patients have consciousness disorders ranging from mild personality changes and behavioral abnormalities to coma.

4) Nervous System Diseases

Acute Phase of Severe Cerebrovascular Diseases: For example, in large - area cerebral infarction, patients have complete paralysis of one - side limbs, speech disorders, accompanied by cerebral edema leading to increased intracranial pressure and even the risk of herniation. Also, with a large amount of intracranial hemorrhage, such as cerebral hemorrhage or subarachnoid hemorrhage, have severe headache, vomiting, consciousness disorders and other symptoms.

Status Epilepticus: Seizures last for more than 30 minutes, or occur frequently with the consciousness not recovering to normal between seizures. Patients show generalized tonic - clonic seizures continuously, which can lead to respiratory depression, cerebral hypoxia and other serious consequences, and even endanger life.

Myasthenia Gravis Crisis: Patients with myasthenia gravis are induced into a crisis due to factors such as infection, surgery, and mental stimulation. They show respiratory muscle weakness, experiencing dyspnea, and in severe cases, require tracheal intubation and mechanical ventilation to maintain breathing.

(2) who had undergone other surgeries within the past six months ;

(3) had comorbidities such as severe rheumatoid arthritis or peripheral neuropathy that could interfere with elbow rehabilitation were excluded;

(4) Individuals with known allergies to any of the medications or materials used during the surgical procedure.

**3.3 Informed Consent**

Before the study began, informed consent was obtained from each and every participant. This process was carried out with great care and thoroughness. The research team provided a detailed explanation of the study's purpose, outlining why the research was being conducted and what it aimed to achieve. They also clearly described the potential risks involved, including not only the risks related to the surgical procedure itself but also those associated with any new or experimental aspects of the study. In addition, the potential benefits were presented to the participants, enabling them to make an informed decision. The consent form was meticulously designed to emphasize the voluntary nature of participation. It clearly stated that the participants had the absolute right to withdraw from the study at any time they wished, without facing any negative consequences or penalties. This was to ensure that the participants felt completely in control of their decision - making process and that their rights were fully protected throughout the study.

**3.4 Early Withdrawal of subjects**

(1)The study protocol included provisions for early withdrawal of subjects if they experienced any adverse events or if their condition worsened.

(2) Participants were also allowed to withdraw from the study if they felt uncomfortable or dissatisfied with the progress of their rehabilitation or the study procedures.

(3) The research team closely monitored all subjects for any signs of distress or complications that might necessitate an early termination of their involvement in the study.

(4) In such cases, the participants were immediately assessed by a medical professional, and appropriate action was taken to ensure their safety and well-being.

(5) The study protocol was designed to prioritize the health and comfort of the participants over the collection of data, reflecting the ethical standards of the research institution.

**3.5 Replacement of participant**

Participant will not be replaced if discontinued.

**3.6 Early termination of the study**

The study was designed with a clear understanding that the well-being of the participants was paramount. In the event that a participant needed to withdraw early, the study protocol was structured to ensure that their health and safety were not compromised. The research team was prepared to provide necessary medical care and support to those who experienced adverse events or worsened conditions. The study's commitment to ethical standards was further demonstrated by the decision not to replace participants who withdrew, as this would have introduced new variables and potentially affected the integrity of the study's results. Moreover, the study was designed with a clause for early termination if it became evident that the research was causing undue harm or if the objectives could not be met without compromising the participants' health. This clause was included to ensure that the study could be stopped at any point if it was no longer in the best interest of the participants or if ethical concerns arose.

**3.7 Interim analyses**

No interim analyses were planned or conducted.


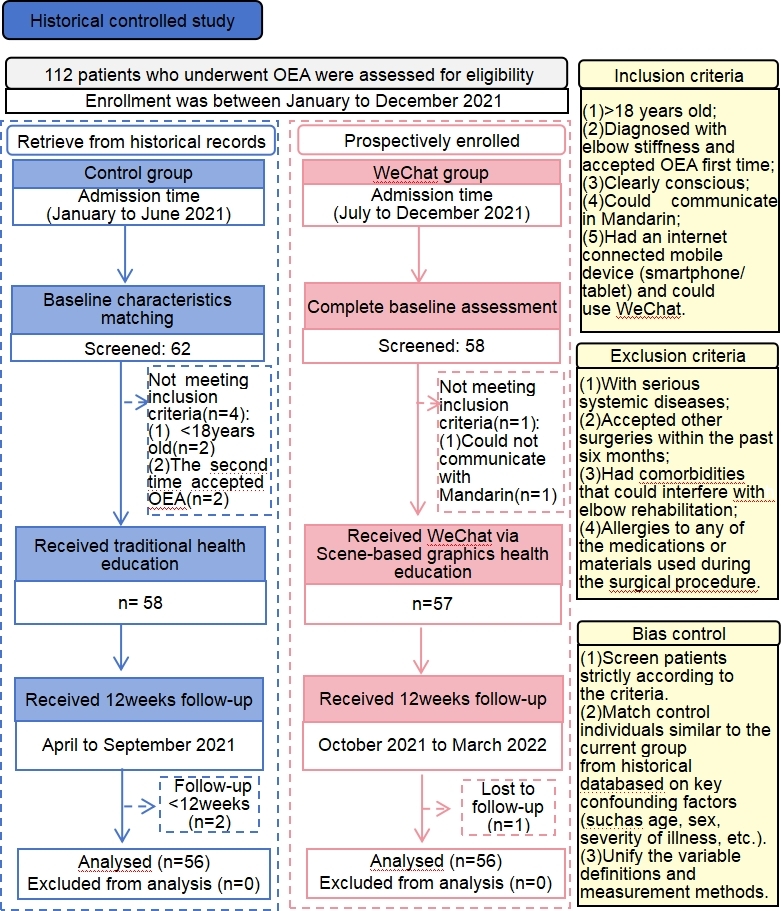


**3.8 Consolidated Standards of Reporting Trials flowchart**

4.**Study Procedures**

The study procedures were meticulously outlined to ensure a systematic approach to data collection and participant management. Each step was designed to be non-invasive and to minimize participant burden, with clear instructions provided to the research staff to follow. The procedures included initial screening, baseline assessments, intervention administration, and follow-up evaluations. All these steps were conducted in a controlled environment to maintain consistency and to reduce the likelihood of external factors influencing the study outcomes. The research team was trained to adhere strictly to the study protocol, ensuring that each participant received the same level of care and attention throughout the study.

4.1**Participant recruitment**

To minimize potential biases, well - trained research nurses in the orthopedic ward delivered recruitment notifications during standardized admission education, strictly adhering to a set script. This script was designed to clearly and comprehensively present the study details.

Patients interested in the study filled an expression - of - interest form, which was collected by a neutral coordinator. This coordinator randomly assigned them to nurses. A trained surgeon - nurse team then evaluated the patients. The evaluation included a physical exam to check for elbow - related signs and the patient's overall condition. Elbow function was assessed using validated tools to obtain objective data on range of motion, strength, and pain. Face - to - face communication was also conducted to understand how the elbow problem affected daily life, providing qualitative insights that complemented the quantitative assessment.

**4.2 Blinding**

No blinding was implemented in this study. The surgical team and research members were aware of group allocations, as the intervention required explicit instructions for the WeChat-based rehabilitation program. Patients were enrolled into different groups at varying admission times to minimize interaction, and all participants were informed about the study procedures without masking group details. Primary outcomes (e.g., elbow range of motion, MEPS scores) were measured using standardized protocols with calibrated instruments, minimizing subjective bias. All data were recorded electronically and analyzed by a statistician unaware of group allocations.

**4.3 Intervention**

The intervention phase was carefully structured to ensure consistency and adherence to the study protocol. Participants were divided into two groups: one receiving the traditional health educatiom and the other receiving the WeChat-based plus scene–graphics health education.

To ensure that the intervention was delivered as intended, regular training sessions were held for the surgeon-nurse teams. These sessions focused on the correct execution of the exercises.

The study protocol also included provisions for participants who experienced adverse events or had difficulty adhering to the intervention. A dedicated support line was established, and participants could reach out to the research team for assistance and guidance at any time during the study.

**4.3.1WeChat group**

1. **A Multidisciplinary Team - based WeChat Health Education Program for Elbow Rehabilitation Intervention**

The multidisciplinary team comprised a surgeon, a nurse manager, a rehabilitation therapist, a platform maintenance specialist, and three elbow joint nurse specialists. Together, they developed a comprehensive health education program. The surgeon was responsible for overseeing the intervention, training team members, and evaluating patients' elbow function. The nurse manager ensured the successful implementation of the intervention, while the rehabilitation therapist created content for elbow rehabilitation and provided instructions on performing the necessary exercises. The three nurse specialists were tasked with delivering the health education.

The platform maintenance specialist was in charge of managing the WeChat group, ensuring its smooth operation, and addressing any technical issues that arose. The group was designed to be interactive, allowing participants to ask questions, share experiences, and receive feedback from the medical professionals involved. This setup aimed to enhance participant engagement and adherence to the intervention program.

The WeChat-based health education program was designed to be user-friendly and accessible, with content tailored to the specific needs of elbow rehabilitation. It included educational materials to reinforce learning and encourage self-management of rehabilitation exercises. The program also incorporated a feature for participants to track their progress and set reminders for their daily exercises, which was intended to promote consistency and accountability.

Throughout the intervention, the multidisciplinary team monitored participant activity within the WeChat group, providing encouragement and answering questions to keep participants motivated and informed. The team also used the group to distribute timely updates on the intervention protocol and to remind participants of upcoming training sessions or support line availability. This approach was designed to maximize the effectiveness of the intervention by fostering a supportive community and ensuring that participants felt supported throughout their rehabilitation journey.

(2)T**he creation, pilot study, and application of a comic book for elbow rehabilitation education in an intervention group with the support of a WeChat group.**

The health education materials used were the same as those used in the control group. Following this, the research team transformed the material into a comic book. Here's how the comic's creation unfolded:

The comic book was crafted with the intention of making the educational content more engaging and easier to understand for the participants. The narrative was designed to follow a character through the rehabilitation process, with each step of the journey illustrating a different aspect of the elbow rehabilitation program. The artwork was vibrant and colorful, capturing the attention of readers and making the information more memorable.

To ensure the comic book was effective, the research team conducted a pilot study with a small group of patients. Feedback from this study was instrumental in refining the content, making sure that the language was appropriate for the target audience and that the illustrations accurately depicted the exercises and techniques being taught.

The final version of the comic book was then distributed to all participants in the intervention group. It was used in conjunction with the WeChat group, where the nurse specialists would reference the comic book during their educational sessions, and participants were encouraged to use it as a reference guide for their home exercises. The comic book became a valuable tool for reinforcing the educational messages and for keeping participants engaged with the rehabilitation program.

(3) **The interactive game for elbow rehabilitation in the WeChat group: design, features and educational role.**

The interactive game was designed to further enhance the learning experience by making it more engaging and interactive. The game was based on the concept of a picture-based cloze test, where participants were presented with images related to elbow rehabilitation and had to fill in the missing words or phrases that described the actions or concepts depicted in the pictures. This approach not only tested the participants' understanding of the rehabilitation process but also reinforced their knowledge in a fun and interactive manner.

The game was first designed as Power point and then integrated into the WeChat group, allowing participants to play it on their smartphones or other devices. The nurse specialists monitored the game's progress and provided immediate feedback and guidance when necessary. This interactive element was particularly effective in keeping the participants motivated and involved in their rehabilitation journey, as it provided a sense of achievement and progress through the game's levels.

The interactive game was designed to provide a fun and engaging way for participants to learn about elbow rehabilitation. It was developed with the aim of making the learning process more interactive and less monotonous. The game incorporated elements of the rehabilitation program, allowing players to virtually experience the exercises and techniques they would encounter in real life.

The game was structured in levels, each level corresponding to a different stage of the rehabilitation process. As players progressed through the game, they unlocked new exercises and challenges, which helped them to understand the importance of each step in their recovery journey.

To further enhance the educational aspect, the game included a progress tracking feature that allowed participants to monitor their improvement over time. This not only motivated them to continue with their rehabilitation but also provided valuable feedback to the nurse specialists, who could tailor their advice and support based on the data collected.

1. **Education process during hospital and after discharge.**

**1)During hospital**

**Step 1: Knowledge memory.**

The research nurse directed patients to access the 'Elbow Special Zone' on the WeChat public account. Patients were then instructed to dedicate fragmented time to self-study the assigned knowledge until they had memorized it. In case of any difficulties, patients were encouraged to consult the research team for guidance.

**Step 2:** **Picture-based cloze test.**

The research nurse conducted a multimedia classroom session in the orthopaedic ward. The session involved the use of PowerPoint to display a comic with blank spaces that patients needed to fill in with correct knowledge. Patients took turns providing answers and were praised for correct responses. For incorrect responses, the nurse provided detailed guidance. Patients who missed the session due to clinical examination received face-to-face guidance.

**Step 3: Error correction.**

Patients were given the opportunity to review their answers and understand the reasons behind any mistakes they had made. This step was crucial for consolidating their learning and preventing the repetition of errors in future exercises. The nurse facilitated a discussion where patients could openly ask questions and express any confusion, ensuring that everyone had a clear understanding before moving on to the next stage of rehabilitation.

**Step 4: Keypoint summary.**

The research nurse summarized the key points and emphasized the common problems discussed in the previous steps. This helped to reinforce the learning and ensure that patients had a comprehensive grasp of the rehabilitation process. The summary was presented in a concise and easy-to-understand manner, often using visual aids to enhance retention.

**Step 5: Motion instruction.**

Patients were provided with a series of motion instructions that were tailored to their specific rehabilitation needs. These instructions were designed to be simple and clear, ensuring that patients could perform the exercises correctly and safely at home. The rehabilitation specialist demonstrated each motion, and patients were encouraged to mimic the movements to practice. To ensure compliance and proper technique, patients were asked to record their exercise sessions and share them with the nurse for feedback. This step was essential for translating the theoretical knowledge into practical skills, which was vital for the patients' recovery.

1. **After discharge**

**Process 1: Rehabilitation check-in.**

Patients were contacted periodically to assess their progress and address any challenges they might be facing with their rehabilitation exercises. This follow-up was conducted via WeChat. The nurse reviewed the recorded exercise sessions and provided constructive feedback, offering encouragement and making adjustments to the exercise regimen as necessary. This ongoing support was instrumental in maintaining patient motivation and ensuring that they adhered to their rehabilitation plan, which was critical for their long-term recovery and independence.

**Process 2: Knowledge consolidation.**

The research nurse utilized Questionnaire Star to design “Picture-based cloze test”.

This test was specifically created to evaluate the patients' understanding of the rehabilitation process and to identify any areas where they might need further clarification or instruction. The picture-based format made it easier for patients to relate to the content, as it depicted real-life scenarios they might encounter during their rehabilitation journey. The results of these tests were then used to tailor subsequent educational materials and reinforce learning, ensuring that patients had a solid foundation of knowledge to support their recovery.

**Process 3: Error correction.**

Patients were given additional resources and materials to further reinforce their understanding of the rehabilitation process. This included detailed documents, and access to an online support group where they could share experiences and tips with others who were also undergoing rehabilitation. The aim was to create a community of support that extended beyond the hospital walls, providing patients with a sense of belonging and ongoing encouragement. The rehabilitation team also organized periodic webinars where patients could ask questions and learn from experts in the field, ensuring that their knowledge remained up-to-date and relevant to their recovery journey.

**Process 4: Answer questions and clarify doubts.**

The rehabilitation team also made themselves available for one-on-one consultations, providing personalized answers to any questions that arose during the rehabilitation process. This level of engagement helped to address concerns promptly and reinforced the patients' confidence in their ability to manage their recovery effectively.

**The processes above were continuous monitoring and feedback by research team.**

To ensure that the rehabilitation process was effective and that patients were progressing as expected, the research nurse implemented a continuous monitoring system. This involved regular check-ins with patients to assess their progress and to identify any challenges they might be facing. The feedback from these sessions was critical in adjusting the rehabilitation plan to better suit the individual needs of each patient. The team also used a feedback loop to incorporate patient suggestions into the program, making it more patient-centered and responsive to their needs. This iterative approach allowed for a dynamic and adaptive rehabilitation process that could evolve with the patient's changing circumstances and recovery milestones.

**4.3.2 Control group**

The control group received traditional health education. Patients were instructed to access an online manual titled 'Elbow Rehabilitation' by scanning a code using WeChat. The manual provided comprehensive information on elbow anatomy, common injuries, and rehabilitation exercises. It was designed to be user-friendly, with clear illustrations and step-by-step instructions to guide patients through their recovery at home. The control group's approach was more self-directed, with less personal interaction compared to the WeChat group, which received the additional support and resources described earlier.

Post-discharge, weekly follow - up calls were made to ensure the continuity of the recovery process. This group's progress was monitored through periodic phone calls, where they were asked to report on their symptoms and adherence to the rehabilitation exercises. The research team used this information to evaluate the effectiveness of the online manual and self-directed approach.

**4.4 Study Duration**

The duration of this study was 15 months.

**4.4.1 Study Outcome**

All datas were collected by using a standardized data collection form and process.

**4.4.1.1Primary Outcome**

The elbow range of motion (ROM) was measured by using a handheld goniometer and consisted of four angles: elbow flexion and extension, forearm pronation, and supination [41, 42]. The ROM was measured three times by a surgeon using three landmarks (the lateral epicondyle, the tip of the acromion process, and the middle portion of the wrist)., after which the average ROM was calculated.

**4.4.1.2 Secondary outcome measures**

The secondary outcomes were elbow function, quality of life and postoperative complications.

1. **Elbow function**

This was assessed using the Mayo Elbow Performance Score (MEPS), a validated scoring system that evaluates pain, range of motion, stability, and daily activities. Patients were scored at the beginning of the study and at the 1-week, 6-weeks and 12-weeks postoperative to determine any improvements.

1. **Quality of life**

This was measured using the was evaluated using the Short Form 36 (SF-36) [41]. Psysiological function was used in this study.

1. **Postoperative complications**

These were recorded and categorized as the formation of heterotopic ossification (HO), new onset or exacerbation of ulnar nerve symptoms, and repeat elbow stiffness. Elbow HO was classified into three grades: Grade 1, no functional limitation; Grade 2A, limited flexion and extension; Grade 2B, limited pronation and supination; Grade 2C, 2A combined with 2B; and Grade 3, ankylosis [43, 45]. The new onset or exacerbation of ulnar nerve symptoms could be observed by using the Dellon classification, which evaluates sensory (paresthesia, vibratory perception, and 2-point discrimination) and motor symptoms (muscle weakness and atrophy) [41,43]. Repeat elbow stiffness could be diagnosed by the ROM of the elbow [41,43].

**4.4.2 Clinical Assessments**

The clinical assessments were conducted by a team of experienced orthopedic surgeons. Any adverse events or complications were documented and managed according to the standard clinical protocols. The data collected from these assessments were then analyzed to evaluate the efficacy and safety of the surgical intervention being studied.

1. **The elbow range of motion (ROM)**

This was measured in degrees, with full extension considered as 0 degrees and full flexion as the maximum angle achieved without pain or discomfort. Pronation and supination were also measured, with the forearm in a neutral position for pronation and fully supinated for supination. The ROM data were then compared to the preoperative measurements to assess the improvement or deterioration in joint function post-surgery. The surgeons also evaluated the presence of pain during motion and its impact on the patient's daily activities. These clinical assessments provided a comprehensive overview of the patient's functional recovery and were crucial in determining the success of the surgical intervention.

1. **Mayo Elbow Performance Score (MEPS)**

The MEPS ranges from 0 to 100, with higher scores indicating better elbow function [42]. The score ranges from 0–100 points and is classified into four categories: excellent (90–100), good (75–89), fair (60–74), and poor (0–59)[11, 41, 42]. The MEPS is calculated based on four parameters: pain, range of motion, stability, and daily activities. Each parameter is assigned a specific number of points, and the total score is then derived from the sum of these points. The assessment of pain is based on the patient's subjective report and the surgeon's objective evaluation during movement. The range of motion is measured as described previously, with the addition of stability assessment, which involves checking for any signs of joint laxity or abnormal movement. Finally, the impact of the patient's elbow condition on their daily activities is evaluated, considering factors such as work, personal care, and recreational activities. The MEPS provides a standardized method for clinicians to quantify the functional outcome of elbow surgery and to communicate this information effectively to patients and other healthcare providers.

1. **Short Form 36 (SF-36)**

This is a self-rating scale consists of eight dimensions: physiological features, physiological function, somatic pain, general health, vitality, social function, affective function, and mental health. The Cronbach's α coefficient ranges from 0.65–0.94. Psysiological function was used in this study. Scores ranged from 0–100, with higher scores indicating better quality of life. The results are compared to norm-based scores to determine the extent of improvement or decline in various aspects of the patient's well-being. This tool is particularly useful in assessing the broader impact of elbow surgery on the patient's life, beyond just the physical function of the joint. By considering both the MEPS and SF-36 outcomes, clinicians can gain a more holistic understanding of the patient's recovery and tailor post-operative care and rehabilitation programs accordingly.

1. **Hastings and Graham classification**

The Hastings and Graham classification is a system that categorizes elbow heterotopic ossification based on the limitation of range of motion: Grade 1, no functional limitation; Grade 2A, limited flexion and extension; Grade 2B, limited pronation and supination; Grade 2C, 2A combined with 2B; and Grade 3, ankylosis [1].

The Hastings and Graham classification, when used in conjunction with the MEPS and SF-36, offers a comprehensive approach to assessing and managing elbow heterotopic ossification, ensuring that patients receive the most appropriate and individualized care.

1. **Dellon classification**

Which evaluates sensory (paresthesia, vibratory perception, and 2-point discrimination) and motor symptoms (muscle weakness and atrophy), provides a detailed framework for assessing nerve function in patients with elbow injuries. The classification system includes three grades, with Grade I indicating minimal sensory or motor impairment and Grade III representing severe impairment. This classification is instrumental in guiding treatment decisions and prognosticating recovery outcomes. By combining the Dellon classification with the Hastings and Graham classification, as well as the functional and quality of life measures provided by the MEPS and SF-36, clinicians can develop a comprehensive treatment plan that addresses both the physical and neurological aspects of the patient's condition. This multidimensional approach ensures that all factors influencing the patient's recovery are taken into account, leading to a more personalized and effective rehabilitation process.

1. **Repeat elbow stiffness**

The repeat elbow stiffness is a common complication that can occur after elbow surgery or trauma. It is characterized by a gradual loss of range of motion and can significantly impact a patient's ability to perform daily activities. The condition is often managed with a combination of physical therapy, medications, and in some cases, surgical intervention. Early identification and treatment are crucial to prevent the progression of stiffness and to optimize functional outcomes. Clinicians may use a variety of assessment tools to monitor the progression of stiffness and to guide treatment decisions. These tools include range of motion measurements, patient-reported outcome measures, and imaging studies to visualize the extent of the stiffness. The goal of managing repeat elbow stiffness is to restore as much function as possible and to minimize pain and discomfort, thereby improving the patient's overall quality of life.

**4.4.3 Statistical Method**

SPSS Statistics version 26.0 (IBM Corp) was utilized for the data analysis. The chi-square test was used to analyze sex, disease side, and the incidence of postoperative complications. The collected data were subjected to a comprehensive statistical analysis to evaluate the effectiveness of the intervention on elbow stiffness. The normality of the data was first assessed using the Shapiro-Wilk test. For variables that satisfied the normality test, parametric statistical methods were employed, including the paired sample t-test for comparing pre-intervention and post-intervention values. For variables that did not meet the normality assumption, non-parametric tests, specifically the Wilcoxon signed-rank test, were utilized. The changes in elbow range of motion (ROM), the Mayo Elbow Performance Score (MEPS), and the Short Form-36 (SF-36) health survey scores at each time point were analyzed using repeated measures analyses of variance (ANOVA). The results were presented as mean ± standard deviation (SD) for continuous variables and as frequencies and percentages for categorical variables. In cases where the data were not normally distributed, median values and interquartile ranges (IQRs) were reported. The level of statistical significance was set at a two-tailed P value < .05. All statistical analyses were performed using appropriate software to ensure accuracy and to provide a robust interpretation of the study findings.

**5.Data**

**5.1 Data Collection**

The data collection process was meticulously designed to ensure the accuracy and reliability of the information gathered. A structured protocol was established to guide the collection of demographic information, medical history, and the specific outcomes of interest. Patients were enrolled in the study following a thorough explanation of the research objectives and the provision of informed consent. Data were collected at multiple time points, including baseline and at 1, 6, and 12 weeks post-operation, to capture the dynamic nature of elbow stiffness and its response to treatment. Standardized forms were used to record range of motion measurements, patient-reported outcomes, and any complications that arose. The data collection team underwent rigorous training to minimize variability and ensure consistency across all measurements. All data were entered into a secure electronic database to facilitate analysis and to protect patient confidentiality.

**5.2 Data Transmission**

The data transmission process was designed with security and efficiency in mind. Each data point was encrypted before being transmitted from the collection site to the central database, ensuring that patient information remained confidential. Secure protocols were used to transfer data over the internet, and access to the database was restricted to authorized personnel only. Regular audits were conducted to monitor data integrity and to ensure compliance with the study's data management plan. Any discrepancies or anomalies identified during the audits were promptly investigated and resolved to maintain the quality of the dataset.

**5.4 Data Storage**

Data storage was carried out using a state-of-the-art database management system that provided robust data backup and recovery solutions. The system was configured to automatically back up data at regular intervals, reducing the risk of data loss due to hardware failure or other unforeseen events. Access to the stored data was strictly controlled through a combination of user authentication and role-based access permissions, ensuring that only authorized personnel could retrieve or modify the information. Furthermore, the database was designed to be scalable, allowing for the seamless integration of additional data as the study progressed. To further safeguard the integrity of the data, a comprehensive audit trail was maintained, documenting all access and changes made to the dataset. This enabled full traceability and accountability for all data-related activities.

**5.5 Data** **Sharing**

Data sharing was conducted in accordance with the highest ethical standards and the study's data sharing policy. Only de-identified data were shared with external collaborators to protect participant privacy. A formal data sharing agreement was established with each collaborator, outlining the terms of data use and ensuring that the data would be used solely for the purposes of the study.

**5.6** **Cancellation of Participation**

In the event of a participant's decision to withdraw from the study, all personal data associated with that individual was immediately removed from the active database. The process was designed to be swift and secure, ensuring that no further use of the participant's information could occur. The participant's data was then archived in a separate, secure location, accessible only for the purpose of maintaining records of the study's conduct and for any follow-up required by the study protocol. This procedure was in place to respect the participant's choice and to maintain the integrity of the research data.

**5.7 Researcher Confidentiality**

Researchers involved in the study were bound by strict confidentiality agreements to protect the sensitive nature of the data and the privacy of the participants. These agreements included clauses that prohibited the unauthorized disclosure of any study-related information to third parties. To further enforce these confidentiality measures, regular training sessions were conducted to educate researchers on best practices for maintaining confidentiality and the potential consequences of non-compliance. The study's leadership also implemented a system of checks and balances to monitor compliance with these confidentiality protocols, ensuring that all research activities were conducted with the utmost discretion and respect for participant privacy.

**5.8 Data Retention Period**

All data were stored securely, with access strictly controlled and monitored to prevent unauthorized access or accidental loss.

1. **Safety**

To ensure participant safety and minimize adverse events, we adhere strictly to national and international ethical norms and regulations, guaranteeing that all research practices comply with both ethical and legal standards.

**6.1 Related to the surgical site**

**6.1.1Severe bleeding or hematoma**

If a large amount of continuous bleeding or a significantly enlarged hematoma is found at the surgical incision site during the rehabilitation process, it may indicate that the damaged blood vessels are not well controlled or that new bleeding points have emerged. Continuing rehabilitation may aggravate the bleeding situation and affect wound healing and joint function recovery.

**6.1.2The aggravation of wound infection**

It is manifested as obvious redness and swelling around the wound, intensified pain, fever, and purulent secretions oozing out, etc. At this time, the body's immune system is mainly focused on fighting the infection. Continuing rehabilitation may lead to the spread of the infection, aggravate the condition and delay healing.

**6.1.3 Structural instability such as fractures or joint dislocations**

It is manifested as obvious redness and swelling around the wound, intensified pain, fever, and purulent secretions oozing out, etc. At this time, the body's immune system is mainly focused on fighting the infection. Continuing rehabilitation may lead to the spread of the infection, aggravate the condition and delay healing.

**6.2 The overall physical condition of the patient**

**6.2.1 Serious systemic adverse reactions**

For example, if a patient has persistent high fever, it may indicate severe infection or other systemic diseases, which can affect their physical strength and recovery ability, making them unfit to continue with rehabilitation treatment.

**6.2.2 Serious cardiovascular problems**

If patients experience cardiovascular system symptoms such as palpitations, chest pain, and breathing difficulties during the rehabilitation process, it may indicate an excessive burden on the heart or the onset of cardiovascular diseases, and rehabilitation needs to be stopped immediately for relevant examinations and treatments.

**6.2.3Extremely weak or tired**

If a patient feels extremely weak and powerless after a period of rehabilitation, unable to complete basic recovery exercises and not improving with rest, this may indicate extreme fatigue or other underlying health issues that require pausing the rehab program for an evaluation.

**6.3Abnormal rehabilitation progress**

**6.3.1 Severely limited joint movement and intensified pain**

If during the rehabilitation process, despite following the plan, the range of joint movement not only fails to improve but decreases sharply, while the degree of pain significantly increases, it may indicate that the rehabilitation method is inappropriate or new intra-articular lesions, such as heterotopic ossification, have emerged. It is necessary to stop the current rehabilitation plan and re-evaluate.

**6.3.2The symptoms of nerve injury worsen**

For example, if the range of sensory abnormalities in the upper limbs (such as numbness and tingling sensation) expands or the muscle strength significantly weakens, it may indicate further nerve damage during the rehabilitation process, and immediate cessation and a neural function assessment are required.

**6.3.3 Unexpected allergic reactions**

In the event that a patient develops an allergic reaction to any of the rehabilitation equipment or materials used, such as skin rashes, itching, or swelling, it is crucial to halt the rehabilitation immediately and seek medical attention. The rehabilitation program should be adjusted to avoid further allergic responses.

**6.3.4 Uncontrolled blood sugar levels**

For patients with diabetes, if blood sugar levels become uncontrollable during rehabilitation, it may be necessary to postpone the rehabilitation process until the blood sugar is stabilized. High or low blood sugar can affect the patient's recovery and overall health, making it unsafe to continue with the rehabilitation exercises.

**6.3.5 Mental health concerns**

If a patient shows signs of increased anxiety, depression, or other mental health issues that interfere with their ability to participate in rehabilitation, it is important to address these concerns before proceeding. Mental health is a critical component of recovery, and psychological support may be required to ensure the patient's safety and progress in rehabilitation.

**6.3.6 Unexplained weight loss or gain**

Significant and unexplained changes in body weight can be a sign of underlying health issues that may affect rehabilitation outcomes. If a patient experiences rapid weight loss or gain, it is advisable to pause the rehabilitation program and investigate the cause before resuming.

**6.3.7 Persistent dizziness or fainting**

If a patient consistently experiences dizziness or fainting spells during rehabilitation sessions, this could indicate a serious issue such as dehydration, low blood pressure, or other cardiovascular problems. Rehabilitation should be stopped, and the patient should be evaluated by a healthcare professional to determine the cause and appropriate course of action.

If have the situations above, the rehabilitation should stopped and change the treatment. In cases where rehabilitation must be halted, it is essential to reassess the patient's condition and modify the rehabilitation plan accordingly. This may involve consulting with specialists, such as endocrinologists for blood sugar issues or mental health professionals for psychological concerns. The revised rehabilitation program should be tailored to the patient's current health status and should only resume once the underlying problem has been resolved and it is deemed safe to do so by a healthcare provider. Regular monitoring and communication with the patient are key to ensuring that the rehabilitation process remains effective and safe throughout the patient's recovery journey.

**6.2 Safety of Data Collection**

**6.2.1 Data encryption and protection**

In the process of collecting patient data, it is crucial to ensure the security and confidentiality of the information. Data encryption should be implemented to protect sensitive information from unauthorized access. Additionally, strict access controls should be in place to ensure that only authorized personnel can view or modify the data.

**6.2.2 Compliance with regulations**

Healthcare providers must adhere to relevant laws and regulations regarding data protection, such as the Health Insurance Portability and Accountability Act (HIPAA) in the United States. Regular audits and compliance checks should be conducted to ensure ongoing adherence to these standards.

**6.2.3 Training for staff**

Staff members involved in data collection and handling should receive comprehensive training on the importance of data security and the proper procedures for protecting patient information. This training should be updated regularly to reflect any changes in regulations or best practices.

**6.2.4 Patient consent and transparency**

Patients should be informed about what data is being collected, how it will be used, and who will have access to it. Obtaining explicit consent from patients for data collection is essential, and they should be given the option to withdraw consent at any time.

**6.2.5 Data integrity and accuracy**

Measures should be in place to ensure the integrity and accuracy of the data collected. This includes regular data validation checks and the implementation of protocols to prevent and correct errors in the data.

By addressing these aspects of data safety, healthcare providers can ensure that the rehabilitation process is not only effective but also secure, maintaining the trust of patients and complying with legal requirements.

**7. Study Monitoring**

**7.1 Regular monitoring and communication**

Communication with the patient are key to ensuring that the rehabilitation process remains effective and safe throughout the patient's recovery journey. This involves keeping detailed records of the patient's progress, any adverse reactions to treatment, and any changes in their overall health status. The rehabilitation team should meet regularly to discuss the patient's case, review the effectiveness of the treatment plan, and make adjustments as necessary.

**7.2 Collect patient's feedback**

The patient's feedback is also an essential component of study monitoring. It is important to solicit and consider the patient's perspective on their treatment and recovery, as this can provide valuable insights into the effectiveness of the rehabilitation program and areas that may need improvement.

By implementing a comprehensive monitoring strategy that includes these various components, healthcare providers can ensure that rehabilitation programs are not only safe and effective but also responsive to the evolving needs of the patient throughout their recovery journey.

**8. Ethical Considerations**

This study was registered with the Chinese Clinical Trial Registry (ChiCTR2000036004) and approved by the Ethics Review Committee of Shanghai Sixth People’s Hospital (Approval Number: 2020–028). Participants were thoroughly briefed on the study's objectives, procedures, potential risks, and anticipated benefits during the enrollment process. Additionally, they were informed about the confidentiality and waiver agreements, which ensured data protection and the right to withdraw from the study without impacting their treatment. Upon comprehending all aspects, participants provided written consent to participate.

**Appendix 1.**

**Informed Consent**

**Title: Comprehensive Health Education for Postoperative Elbow Rehabilitation**

**Informed Consent • Informed Notice page**

Dear participant,

We are conducting a study to evaluate the effectiveness of a new comprehensive health education program to improve rehabilitation outcomes after open elbow arthrolysis. Participation in this study is completely voluntary, and you may choose to withdraw at any time without any negative consequences to your care.

The rehabilitation strategy outlined in this document is designed to help you regain the full range of motion and strength in your elbow after surgery. It includes a series of exercises and activities that you will need to perform regularly. The program is tailored to your specific needs and progress will be monitored closely by our rehabilitation team.

Please be aware that there are certain precautions you must take to ensure a safe and effective recovery.

1. The program emphasizes the importance of gradual progression in exercise intensity to prevent overexertion and injury.

2. It is crucial to follow the rehabilitation schedule as closely as possible to achieve optimal results.

3. The rehabilitation team will provide regular feedback and adjustments to the program based on your performance and recovery status.

4. The strategy also includes educational materials to help you understand the healing process and the significance of each exercise.

5. Adherence to the precautions outlined in this document is essential for a successful recovery and to minimize the risk of complications.

Remember, the ultimate goal of the rehabilitation program is to help you return to your daily activities and hobbies with minimal limitations. Your active participation and adherence to the program are vital to this success. We are here to support you every step of the way, and together, we will work towards your full recovery. We will keep your personal information confidential. All your data will be used solely for this study and will not be used for other purposes or disclosed.

If you have any questions or concerns, please do not hesitate to contact the research team at the provided phone number(021-3829-7726) or email address (Yin1230102@163.com). We are committed to ensuring your comfort and safety throughout this process.

**Introduction**

Normal elbow mobility is essential for performing daily activities efficiently. It is a fundamental aspect that allows individuals to engage in a variety of tasks effortlessly. Unfortunately, elbow stiffness is a common issue, affecting up to 56% of individuals following elbow trauma. This condition is characterized by a significant restriction in the range of motion or rotation of the elbow joint, impeding the ability to perform everyday activities, including dressing, eating, working, and engaging in recreational activities. Open elbow arthrolysis (OEA) is the most frequently used surgical intervention for this condition. Research and clinical practice have demonstrated its effectiveness in restoring elbow function and range of motion. During the procedure, surgeons remove bony obstructions or scar tissue within the olecranon fossa to achieve optimal flexion and extension of the elbow joint.

Postoperative rehabilitation is crucial for the successful recovery of elbow function. Timely and appropriate rehabilitation leads to favorable outcomes, whereas inadequate rehabilitation can result in serious complications. These complications may include heterotopic ossification (HO), ulnar nerve symptoms, and recurrent elbow stiffness, all of which can significantly impact the patient's quality of life and necessitate further surgical interventions. Statistics indicate that postoperative elbow issues occur in 56-87% of cases, with the likelihood of recurrent stiffness ranging from 8.4-47%. Secondary surgeries are required in 10-34% of cases, and patient dissatisfaction due to these issues reaches 21%. These figures highlight the necessity for precise and effective postoperative rehabilitation. Standard postoperative rehabilitation typically spans one year and is divided into three phases: the acute phase (first day to 6 weeks), the subacute phase (6 weeks to 3 months), and the functional phase (3 months to one year). Each phase involves specific rehabilitation exercises designed to progressively restore elbow function.

It is important to recognize that in addition to the initial rehabilitation process that takes place within the hospital environment, patients are also required to continue their rehabilitation efforts at home. This home - based rehabilitation is an integral part of the overall recovery process. As a result, having a comprehensive and highly accurate grasp of the relevant knowledge related to rehabilitation is absolutely crucial for ensuring effective rehabilitation outcomes.

**Research Purpose：**

**Primary Objective：**

To evaluate the efficacy of a comprehensive health education program in enhancing patient rehabilitation exercises post-elbow surgery.

**Secondary Objective:**

1. To assess the impact of the health education program on the quality of life and elbow function.

1. To assess the impact of the health education program on the reduction of postoperative complications such as heterotopic ossification and ulnar nerve symptoms.
2. To evaluate the program's influence on the rate of recurrent elbow stiffness and the need for secondary surgeries.

**Inclusion and exclusion criteria.**

**Inclusion criteria**

1. older than 18 years old,
2. diagnosed with elbow stiffness,
3. had undergone open elbow arthrolysis the first time,
4. clearly conscious(Glasgow Coma Scale score of 15),
5. could communicate with a researcher in Chinese (Mandarin) normally,
6. had a mobile device (e.g., smartphone or tablet) with an internet connection and were capable of using WeChat.

**exclusion criteria**

1. with serious systemic diseases (e.g., uncontrolled hypertension with a blood pressure consistently above 180/110 mmHg, poorly controlled diabetes with HbA1c > 9%, or active malignancy )

Here are some systemic diseases that should be excluded in this study:

1.1 Cardiovascular System Diseases

1.1.1 Severe Coronary Heart Disease: Such as having had a myocardial infarction recently, or suffering from severe unstable angina where angina pectoris occurs frequently even at rest, and there are multiple severe coronary artery stenoses.

1.1.2 Severe Cardiac Arrhythmia: For example, sustained ventricular tachycardia, a history of ventricular fibrillation, or sick sinus syndrome without a pacemaker installed, resulting in patients often experiencing dizziness, blackouts or even syncope.

1.1.3 Severe Heart Failure: Like patients with New York Heart Association (NYHA) class Ⅲ - Ⅳ heart failure, presenting with obvious dyspnea that occurs even with slight activity or at rest, accompanied by peripheral edema, hepatomegaly and other symptoms of systemic congestion.

1.2 Respiratory System Diseases

1.2.1 Acute Exacerbation of Severe Chronic Obstructive Pulmonary Disease (COPD): Patients experience severe dyspnea, along with increased respiratory rate, use of accessory muscles for breathing. Blood gas analysis shows severe hypoxemia and/or hypercapnia, such as arterial partial pressure of oxygen (PaO₂) being lower than 50 mmHg and partial pressure of carbon dioxide (PaCO₂) being higher than 50 mmHg.

1.2.2 Status Asthmaticus: Refers to a severe asthma attack that does not relieve after conventional treatments such as bronchodilators, lasting for more than 24 hours. Patients show extreme dyspnea, orthopnea, inability to speak in complete sentences, and widespread wheezing in both lungs can be heard. In severe cases, the wheezing may weaken or disappear due to severe airway obstruction (silent chest).

1.2.3 Acute Respiratory Distress Syndrome (ARDS): Usually induced by severe infections, trauma and other factors. Patients have progressive dyspnea, a significant decrease in the oxygenation index (PaO₂/FiO₂), generally less than 200 mmHg, and chest imaging shows diffuse infiltrates in both lungs.

1.3 Digestive System Diseases

1.3.1 Acute Severe Pancreatitis: Patients have severe upper abdominal pain that can radiate to the lower back, accompanied by nausea and vomiting. Examinations may reveal a significant increase in serum amylase and lipase, often more than three times the upper limit of normal. Meanwhile, multiple organ dysfunction syndrome (MODS) may occur, such as acute kidney injury, respiratory failure, etc.

1.3.2 Decompensated Cirrhosis of the Liver with Severe Complications: Such as the presence of massive ascites causing abdominal distension, esophageal and gastric variceal bleeding manifested as hematemesis and/or melena, hepatic encephalopathy where patients have consciousness disorders ranging from mild personality changes and behavioral abnormalities to coma.

1.4 Nervous System Diseases

1.4.1 Acute Phase of Severe Cerebrovascular Diseases: For example, in large - area cerebral infarction, patients have complete paralysis of one - side limbs, speech disorders, accompanied by cerebral edema leading to increased intracranial pressure and even the risk of herniation. Also, with a large amount of intracranial hemorrhage, such as cerebral hemorrhage or subarachnoid hemorrhage, have severe headache, vomiting, consciousness disorders and other symptoms.

1.4.2 Status Epilepticus: Seizures last for more than 30 minutes, or occur frequently with the consciousness not recovering to normal between seizures. Patients show generalized tonic - clonic seizures continuously, which can lead to respiratory depression, cerebral hypoxia and other serious consequences, and even endanger life.

1.4.3 Myasthenia Gravis Crisis: Patients with myasthenia gravis are induced into a crisis due to factors such as infection, surgery, and mental stimulation. They show respiratory muscle weakness, experiencing dyspnea, and in severe cases, require tracheal intubation and mechanical ventilation to maintain breathing.

1. had undergone other surgeries within the past six months
2. had comorbidities such as severe rheumatoid arthritis or peripheral neuropathy that could interfere with elbow rehabilitation were excluded.

**How many people will participate in this study?**

Our institution aims to recruit 58 participants for this study. If you interested in it,

you can contact us at the following address or phone number.

**Study Procedure**

The study will be conducted over a period of 12 weeks, during which participants will undergo a series of tests and evaluations to assess the effectiveness of the rehabilitation program.

(1)Before enroll into the study, the comprehensive medical evaluation of each participant to establish a baseline for their health status. Make sure you don’t have any systemic diseases we have mentioned above.

(2)The study will be conducted in two phases.

1)The first phase was in hospital, involve the self leaning for elbow rehabilitation and undergoing rehabilitation under the supervision of medical professionals. In this step, you will gain materials from the research team through scan the QR code. You can learn the materials by yourself and any doubt can ask the research team. After surgery, you will have the rehabilitation under the instruction by the research team every morning. This phase lasts one week.

2)The second phase will be carried out after discharge, focusing on the continuation of rehabilitation at home and regular follow-up visits to monitor the progress and any adverse effects. The materials in the first phase will be provided to participants for home use, ensuring they have the necessary tools and information to continue their rehabilitation effectively. Participants will be instructed on how to use these materials and will be given a schedule for their home exercises. The follow-up visits will be scheduled at regular intervals to assess the participant's progress and to make any necessary adjustments to their rehabilitation program. During these visits, the medical team will also check for any signs of complications or adverse effects that may have arisen since the last visit. The overall goal of the study is to evaluate the effectiveness of the rehabilitation program and to determine if the combination of supervised and home-based rehabilitation can lead to improved outcomes for patients recovering from elbow surgery.

**Additional information**

**1. Risks and/or discomforts of participating in this study**

Participation in this study posed no risks. However, you may experience some discomfort due to the rehabilitation exercises, which is normal and expected. It is important to communicate any pain or discomfort to the medical team immediately. The research team will provide guidance on how to manage such discomfort and ensure that the exercises are performed safely and effectively. Participants will also be advised on how to recognize signs of overexertion or complications that may require medical attention. The study aims to minimize any potential risks by closely monitoring participants throughout the rehabilitation process and adjusting the program as needed.

1. **Benefits of participating in the study**

Participants may benefit from the study by receiving personalized attention and care from the medical team, which can lead to a more tailored rehabilitation experience. The home-based portion of the program allows for flexibility and convenience, enabling participants to integrate exercises into their daily routine without the need for frequent visits to a medical facility. This can potentially lead to better adherence to the rehabilitation regimen and, consequently, improved recovery outcomes. Additionally, participants will have access to the latest rehabilitation techniques and materials, which may not be available outside of a research setting. By contributing to the study, participants also play a crucial role in advancing medical knowledge and helping future patients who undergo similar surgical procedures.

1. **Alternative treatment options if not participating in the study**

If you choose not to participate in the study, you will still have access to standard rehabilitation care provided by your healthcare provider. This typically includes a series of exercises and physical therapy sessions that are designed to help you regain strength and mobility in your elbow. Standard care may not be as personalized or as frequently monitored as the care provided in the study, and it may not include the latest rehabilitation techniques or materials. However, it is still an effective way to recover from elbow surgery and can lead to a successful outcome. It is important to discuss with your healthcare provider the best rehabilitation plan for your specific needs and circumstances.

1. **Research-related updates**

Participants will be kept informed about the progress and findings of the study through regular updates. These updates will provide insights into how the rehabilitation program is evolving and how the data collected is contributing to the broader understanding of post-surgical recovery. The research team will ensure that all participants are aware of any new developments that may affect their involvement or the potential benefits they can expect from the study. This transparency helps maintain trust and encourages continued participation throughout the duration of the research.

1. **Cost for participate this study**

The study is conducted at no cost to the participants. All expenses related to the rehabilitation program, including any materials and techniques used, are covered by the research funding. This means that individuals who join the study do not have to bear any financial burden that might otherwise be associated with accessing advanced rehabilitation care. The study aims to provide the highest standard of care without any out-of-pocket costs for the participants, ensuring that the focus remains on recovery and contributing to medical science.

1. **Confidentiality and privacy**

Confidentiality and privacy measures will be strictly enforced to protect participants' personal information. The research team will use coded identifiers instead of names to maintain anonymity. All data will be stored securely and only accessible to authorized personnel involved in the study. Participants will be informed about their rights, including the right to withdraw from the study at any time without penalty. The study will comply with all ethical guidelines and regulations to ensure the safety and well-being of all participants.

1. **Right and responsibility**

1)Right :

Participants have the right to be fully informed about the study, including its purpose, procedures, potential risks, and benefits. They also have the right to ask questions and receive answers in a timely manner. The right to consent or refuse participation is fundamental, and participants can withdraw from the study at any point without any negative consequences. Additionally, participants have the right to receive any new information that may affect their decision to continue in the study.

2)Responsibility :

Participants are responsible for providing accurate and complete information about their health status and any changes that occur during the course of the study. They are expected to follow the study's procedures, attend scheduled appointments, and report any adverse events or concerns promptly. Cooperation with the research team is essential for the integrity of the study and the safety of all participants.

1. **Attention**

Should you encounter any of the following scenarios, please do not hesitate to reach out to us. You may require alternative treatments and should discontinue rehabilitation.

**8.1** **Related to the surgical site**

**8.1.1****Severe bleeding or hematoma**

If a large amount of continuous bleeding or a significantly enlarged hematoma is found at the surgical incision site during the rehabilitation process, it may indicate that the damaged blood vessels are not well controlled or that new bleeding points have emerged. Continuing rehabilitation may aggravate the bleeding situation and affect wound healing and joint function recovery.

**8.1.2****The aggravation of wound infection**

It is manifested as obvious redness and swelling around the wound, intensified pain, fever, and purulent secretions oozing out, etc. At this time, the body's immune system is mainly focused on fighting the infection. Continuing rehabilitation may lead to the spread of the infection, aggravate the condition and delay healing.

**8.1.3** **Structural instability such as fractures or joint dislocations**

It is manifested as obvious redness and swelling around the wound, intensified pain, fever, and purulent secretions oozing out, etc. At this time, the body's immune system is mainly focused on fighting the infection. Continuing rehabilitation may lead to the spread of the infection, aggravate the condition and delay healing.

**8.2 The overall physical condition of the patient**

**8.2.1** **Serious systemic adverse reactions**

For example, if a patient has persistent high fever, it may indicate severe infection or other systemic diseases, which can affect their physical strength and recovery ability, making them unfit to continue with rehabilitation treatment.

**8.2.2** **Serious cardiovascular problems**

If patients experience cardiovascular system symptoms such as palpitations, chest pain, and breathing difficulties during the rehabilitation process, it may indicate an excessive burden on the heart or the onset of cardiovascular diseases, and rehabilitation needs to be stopped immediately for relevant examinations and treatments.

**8.2.3Extremely weak or tired**

If a patient feels extremely weak and powerless after a period of rehabilitation, unable to complete basic recovery exercises and not improving with rest, this may indicate extreme fatigue or other underlying health issues that require pausing the rehab program for an evaluation.

**8.3Abnormal rehabilitation progress**

**8.3.1** **Severely limited joint movement and intensified pain**

If during the rehabilitation process, despite following the plan, the range of joint movement not only fails to improve but decreases sharply, while the degree of pain significantly increases, it may indicate that the rehabilitation method is inappropriate or new intra-articular lesions, such as heterotopic ossification, have emerged. It is necessary to stop the current rehabilitation plan and re-evaluate.

**8.3.2****The symptoms of nerve injury worsen**

For example, if the range of sensory abnormalities in the upper limbs (such as numbness and tingling sensation) expands or the muscle strength significantly weakens, it may indicate further nerve damage during the rehabilitation process, and immediate cessation and a neural function assessment are required.

**Informed Consent Form • Consent Signature Page**

The informed consent form is a crucial document that outlines the details of the rehabilitation program, including the potential risks and benefits. By signing the consent form, you acknowledge that you have been fully informed about the nature of the program and agree to participate voluntarily. It is important to read the form carefully before signing and to ask any questions you may have to ensure you understand all aspects of the program. Your signature on the consent form signifies your agreement to follow the program guidelines and to participate in the research study. Remember, your health and safety are our top priorities, and we are dedicated to providing you with the best possible care during your recovery journey.

If you agree to participate in this study, please sign below. Thank you once again for your trust and cooperation!

Participant Signature: Date:

Contact Phone Number:

**Appendix 2.**

**Elbow rehabilitation protocol**

This program lasts for one year and the exercise are supervised by research team.

It must adhere to the principle of proceeding step-by-step, combining dynamic and static exercises, and integrating active and passive approaches. The program is divided into three distinct phases.

(1)Rehabilitation－related motions

1)The first phase (the first day to 6weeks post-operative):

I. Fist pump exercise:

This simple exercise helps to increase blood circulation in the hand and forearm, preparing the muscles for more intense elbow movements. Patients are instructed to make a gentle fist and then release it repeatedly. This action should be done slowly and smoothly to avoid sudden jerks.

II. Passive elbow flexion and extension motions:

In this exercise, a trained therapist or a family member with proper guidance assists the patient. The patient relaxes the elbow muscles completely while the helper gently moves the elbow joint to its maximum flexion and extension within a pain - free range. With the goal of maintaining joint mobility and preventing stiffness.

III. Assisted elbow flexion and extension motions:

Similar to the passive motions, but here the patient starts to participate slightly. The patient uses the muscles around the elbow with the help of the assistant to achieve flexion and extension. This collaborative effort helps the patient regain control over the elbow movement gradually.

IV. Active elbow flexion and extension motions:

The patient now performs the flexion and extension movements independently, using their own muscle strength. It is crucial to maintain proper form during these movements. The patient should focus on smooth and controlled motions.

V. Forearm rotation exercises:

With the elbow bent at a 90 - degree angle, the patient rotates the forearm clockwise and counterclockwise. This exercise helps to improve the rotational function of the elbow joint.

2)The second phase (6 weeks to 12 weeks post-operative):

1. Shoulder circular rotation exercise:

This exercise is added to involve the shoulder joint and maintain its flexibility. The patient rotates the shoulder in a circular motion, both forward and backward. This helps to prevent compensatory issues in the shoulder due to elbow problems.

1. Passive elbow flexion and extension motions:

During this phase, the range of motion and the intensity of the passive motions may be adjusted based on the patient's progress. The therapist may increase the flexion and extension angles slightly, but still within a comfortable range for the patient.

1. Assisted elbow flexion and extension motions:

The assistance provided in this phase is reduced as the patient's strength and control improve. The focus is on refining the movement pattern and increasing the patient's confidence in using the elbow.

1. Active elbow flexion and extension motions:

The patient continues to perform these movements independently, but now with more strength and control. The number of repetitions may be increased, and the patient can start to add a slight resistance by using a light elastic band if possible.

1. Forearm rotation exercises:

Similar to the first phase, but the speed and range of rotation can be increased to further enhance the forearm's rotational ability.

3)The third phase (12 weeks to 1 year post-operative):

The exercises are the same as the second phase in terms of types. However, strength training is introduced. For example, the patient can use light weights starting from 1kg. In the passive elbow flexion and extension motions, the therapist may apply a gentle force against the movement to challenge the joint stability and muscle strength. During the assisted and active motions, the use of weights can be incorporated to make the movements more challenging. For the forearm rotation exercises, weighted cuffs can be used to increase the resistance during rotation. This phase aims to build muscle strength and endurance to support the long - term functionality of the elbow joint. As the patient progresses, the weight can be gradually increased under the supervision of a healthcare professional.

1. Rehabilitation - related Attention
2. The patient should be monitored closely for any signs of pain or discomfort during these exercises.
3. It is crucial to ensure that the patient does not overexert themselves, as this could lead to setbacks in the recovery process.
4. The therapist will provide guidance on proper form and technique to prevent injury and to maximize the effectiveness of the exercises.
5. Regular assessments will be conducted to track the patient's progress and to adjust the rehabilitation program accordingly.
6. It is also important for the patient to maintain consistency with the exercises, as this will contribute to a more successful and sustainable recovery.

**Appendix 3.**

Sample table 1 Contents and characters of Scene-based graphics

| Section | Item | Content | Design feature |
| --- | --- | --- | --- |
| First acquaintance  of elbow | **·**Dissection of elbow | **·**Elbow composition | **·**Anatomical picture and Comic, use different colors present different tissues |
|  | **·**Function of elbow | **·**Range of motion  **·**Daily activity | **·**Arrows to indicate the motion direction and mark the Angle value  **·**Indicate daily activities of elbow by comic |
| Recovery Song | **·**Action points | **·**①ROM exercises^a^  **·**②Strength exercise^a^  **·**③Joint draft^a^  **·**④neurorehabilitation^b^ | **·**Breakdown diagram for each action,  arrows and dotted lines mark motion direction  **·**The location, tools, and frequency of the exercise are represented by comic  **·**Put the rehabilitation formula into the painting, such as "1 loose 2 move 3 ice and 4 fixed, if there is severe pain, do not force." |
|  | **·**Rehabilitation notes | **·**common problem process | **·**indicate by warning symbol |
| Rehabilitation "Double-edged Sword" | **·**Hinged external fixation | **·**instructions of structure, pros and cons,adjustment | **·**Illustrate the function of each component, pros and cons  **·**Adjustment tension and direction marked by cirle and arrow |
| Advance in spite of difficulties | **·**Complications judgment and management | **·**Surgery complications  **·**Rehabilitation complications | **·**Marked by symbols,asnausea,dizziness  **·**Marked by different symbols,as Pin site infection, new onset or exacerbation of ulnar nerve symptoms |

^a^: the motions which should be completed postoperative 0-12 weeks

^b^:the motions which should be completed Postoperative 0-3 weeks

Sample table 2 Study schedule and process for daily class

| Study schedule | | | | | |
| --- | --- | --- | --- | --- | --- |
| Stage | Contents | | | Correspond block | |
| Preoperative 1 day | **·**Elbow anatomy and function | | | **·**First acquaintance of elbow  **·**Advance in spite of difficulties | |
| Postoperative 1 day | **·**Hinged external fixation introduction  **·**Action points (Postoperative 0-3 weeks 1) | | | **·**Rehabilitation "Double-edged Sword"  **·**Recovery Song | |
| Postoperative 2 day | **·**Pain coping 1  **·**Action points (Postoperative 0-3 weeks 2) | | | **·**Advance in spite of difficulties  **·**Recovery Song | |
| Postoperative 3 day | **·**Pain coping 2  **·**Action points (Postoperative 3-6 weeks 1) | | | **·**Advance in spite of difficulties  **·**Recovery Song | |
| Postoperative 4 day | **·**Action points (Postoperative 3-6 weeks 2)  **·**Rehabilitation complications treatment | | | **·**Advance in spite of difficulties  **·**Recovery Song | |
| Postoperative 5 day | **·**Action points (Postoperative 6-12 weeks 1)  **·**Action points (Postoperative 6-12 weeks 2) | | | **·**Recovery Song  **·**Recovery Song | |
| Process for daily class | | | | | |
| Step | | Method | Mode | | Time |
| Knowledge memory | | **·**Self-study | **·**WeChat platform | | Fragmented time |
| Look at the picture and fill in the words | | **·**Collective interaction  **·**One-to-one instruction | **·**PowerPoint | | 15 minutes |
| Error correction | | **·**Collective interaction  **·**One-to-one instruction | **·**Paper document  **·**Oral explanation | | 10 minutes |
| Key points summary | | **·**Collective interaction  **·**One-to-one instruction | **·**Paper document  **·**Oral explanation | | 10 minutes |
| Motion instruction | | **·**One-to-one instruction | **·**Action demonstration | | 15 minutes |

References

1. Liu W, Sun Z, Xiong H, Liu J, Lu J, Cai B,et al. Development and validation of a prognostic nomogram for open elbow arthrolysis: the Shanghai Prediction model for Elbow Stiffness Surgical Outcome. Bone Joint J. 2022;104-B(4):486-494.
2. Zheng W, Liu J, Song J, Fan C.. Risk factors for development of severe post-traumatic elbow stiffness. Int Orthop 2018; 42: 595–600.
3. Sun Z, Liu W, Li J, Fan C. Open elbow arthrolysis for post-traumatic elbow stiffness: an update. Bone Jt Open. 2020;1(8):576-584.
4. Siemensma MF, van der Windt AE, van Es EM, Colaris JW, Eygendaal D. Management of the stiff elbow: a literature review. EFORT Open Rev. 2023;8(5):351-360.

5. Zhang D, Nazarian A, Rodriguez EK. Post-traumatic elbow stiffness: Pathogenesis and current treatments. Shoulder Elbow. 2020;12(1):38-45.

1. Sun Z, Cui H, Liang J, Li J, Wang X, Fan C. Determining the effective timing of an open arthrolysis for post-traumatic elbow stiffness: a retrospective cohort study. BMC Musculoskelet Disord. 2019;20(1):122-130.

7. Cui H, Sun Z, Ruan J, Yu Y, Fan C. Effect of enhanced recovery after surgery (ERAS) pathway on the postoperative outcomes of elbow arthrolysis: A randomized controlled trial. Int J Surg. 2019;68:78-84.

8. Masci G, Cazzato G, Milano G, Ciolli G, Malerba G, Perisano C, et al. The stiff elbow: Current concepts. Orthop Rev (Pavia). 2020; 12(Suppl 1):8661-8666.

9. G. Vasileiadis,Taghi Ramazanian,Saygın Kamacı,Daniel R. Bachman,Sang Eun Park,Sutee Thaveepunsan, et al. Loss of pronation-supination in patients with heterotopic ossification around the elbow. J Shoulder Elbow Surg. 2019;28(7):1406-1410.

10. Siemensma MF, van der Windt AE, van Es EM, Colaris JW, Eygendaal D. Management of the stiff elbow: a literature review. EFORT Open Rev. 2023;8(5):351-360.

11. Ruan JH, Cui HM, Sun ZY, Chen S, Wang W, Fan CY. Midterm Outcomes After Open Arthrolysis for Posttraumatic Elbow Stiffness in Children and Adolescents. J Pediatr Orthop. 2021;41(3):e266-e271.

12. Guglielmetti CLB, Gracitelli MEC, Assunção JH, Andrade-Silva FB, Pessa MMN, Luzo MC, et al. Randomized trial for the treatment of post-traumatic elbow stiffness: surgical release vs. rehabilitation. J Shoulder Elbow Surg. 2020; 29(8):1522-1529.

13. Kwak J-M, Sun Y, Kholinne E, Koh K-H, Jeon I-H. Surgical outcomes for posttraumatic stiffness after elbow fracture: comparison between open and arthroscopic procedures for intra- and extra-articular elbow fractures. J Shoulder Elbow Surg. 2019; 28(10):1998–2006.

1. SunZ. 肘关节僵硬新分型和功能评分的建立及远期松解疗效的探究[Establishment of a new classification and functional score for elbow stiffness and exploration of the long - term efficacy of release]. Shanghai Jiao Tong University. 2020.
2. Porcellini, G, Rotini, R, Kantar, SS, Giacomo, SD.The Elbow: Principles of Surgical Treatment and Rehabilitation.Cham: Springer International; 2018.
3. Haglin JM, Kugelman DN, Christiano A, Konda SR, Paksima N, Egol KA. Open surgical elbow contracture release after trauma: results and recommendations. J Shoulder Elbow Surg. 2018; 27(3):418-426.

17. Debono B, Wainwright TW, Wang MY, Sigmundsson FG, Yang MMH, Smid-Nanninga H, et al. Consensus statement for perioperative care in lumbar spinal fusion: Enhanced Recovery After Surgery (ERAS®) Society recommendations. Spine J. 2021;21(5):729-752.

18. Gustafsson UO, Scott MJ, Hubner M, Nygren J, Demartines N, Francis N, et al. Guidelines for Perioperative Care in Elective Colorectal Surgery: Enhanced Recovery After Surgery (ERAS®) Society Recommendations: 2018. World J Surg. 2019 Mar;43(3):659-695.

19. Absolom K, Warrington L, Hudson E, Hewison J, Morris C, Holch P, et al. Phase III Randomized Controlled Trial of eRAPID: eHealth Intervention During Chemotherapy. J Clin Oncol. 2021;39(7):734-747.

20. Xu L, Li P, Hou X, Yu H, Tang T, Liu T, et al. Middle-aged and elderly users' continuous usage intention of health maintenance-oriented WeChat official accounts: empirical study based on a hybrid model in China. BMC Med Inform Decis Mak. 2021;21(1):257-267.

21. Wang J, Wu L. A comparison of health communication effectiveness and the improvement of management strategies: taking two Chinese traditional medicine hospitals' WeChat public accounts as examples. BMC Health Serv Res. 2020; 20(1):1055-1062.

22. Song J, Ma L. Effect of cognitive behavioral therapy and WeChat-based health education on patients underwent peripherally inserted central catheter line placement. Am J Transl Res. 2021;13(12):13768-13775.

23. Sun M, Yang L, Chen W, Luo H, Zheng K, Zhang Y,et al. Current status of official WeChat accounts for public health education. J Public Health (Oxf). 2021;43(3):618-624.

24. Li Y, Xiao QL, Li M, Zhang Y, Chen M, Jiang CH, et al. Community-based intervention via WeChat official account to improve parental health literacy among primary caregivers of children aged 0 to 3 years: Protocol for a cluster randomized controlled trial. Front Public Health. 2023; 6(10):1039394.

25. Tencent . Tencent Announces 2020 Fourth Quarter and Annual Results 2021 Available online at: [https://www.tencent.com/en-us/investors/financial-news.html](https://www.tencent.com/en-us/investors/financial-news.html) (October 10, 2021).

1. Ma X, Lu J, Liu W. Influencing Factors on Health Information to Improve Public Health Literacy in the Official WeChat Account of Guangzhou CDC. Front Public Health. 2021; 3(9):657082.
2. Zhang X, Xiao H, Chen Y. Evaluation of a WeChat-based life review programme for cancer patients: A quasi-experimental study. J Adv Nurs. 2019 Jul;75(7):1563-1574.
3. Zhou K, Wang W, Zhao W, Li L, Zhang M, Guo P, et al. Benefits of a WeChat-based multimodal nursing program on early rehabilitation in postoperative women with breast cancer: A clinical randomized controlled trial. Int J Nurs Stud. 2020;106:103565.

29. Cao Y, Lin SH, Zhu D, Xu F, Chen ZH, Shen HH, Li W. WeChat Public Account Use Improves Clinical Control of Cough-Variant Asthma: A Randomized Controlled Trial. Med Sci Monit. 2018; 24:1524-1532.

30. Jiang Y, Liu F, Guo J, Sun P, Chen Z, Li J, Cai L, Zhao H, Gao P, Ding Z, Wu X. Evaluating an Intervention Program Using WeChat for Patients With Chronic Obstructive Pulmonary Disease: Randomized Controlled Trial. J Med Internet Res. 2020;22(4):e17089.

31. Li T, Ding W, Li X, Lin A. Mobile health technology (WeChat) for the hierarchical management of community hypertension: protocol for a cluster randomized controlled trial. Patient Prefer Adherence. 2019;13:1339-1352.

32. Li Q, Guo L, Zhang J, Zhao F, Hu Y, Guo Y, Du X, Zhang S, Yang X, Lu C. Effect of School-Based Family Health Education via Social Media on Children's Myopia and Parents' Awareness: A Randomized Clinical Trial. JAMA Ophthalmol. 2021;139(11):1165-1172.

33. Ma C, Wang B, Zhao X, Fu F, Zheng L, Li G, Guo Q. WeChat-based education and rehabilitation program in unprotected left main coronary artery disease patients after coronary artery bypass grafting: an effective approach in reducing anxiety, depression, loss to follow-up, and improving quality of life. Braz J Med Biol Res. 2021;54(4):e10370. .

1. Isaac M. Role of humanities in modern medical education. Curr Opin Psychiatry. 2023;36(5):347-351.
2. Chernick LS, Konja A, Gonzalez A, Stockwell MS, Ehrhardt A, Bakken S, et al. Designing illustrative social media stories to promote adolescent peer support and healthy sexual behaviors. Digit Health. 2022;8:1-12.
3. Hall CE, Hall AB, Mallya J, Courtright P, Kok G. Developing comic strips promoting diabetic retinopathy screening in Kilimanjaro, Tanzania, using Intervention Mapping. Eye (Lond). 2022;36(Suppl 1):25-32.
4. Haan MM, van Gurp JL, Knippenberg M, Olthuis G. Facilitators and barriers in using comics to support family caregivers of patients receiving palliative care at home: A qualitative study. Palliat Med. 2022;36(6):994-1005.
5. De Stefano A, Rusciano I, Moretti V, Scavarda A, Green MJ, Wall S, et al. Graphic medicine meets human anatomy: The potential role of comics in raising whole body donation awareness in Italy and beyond. A pilot study. Anat Sci Educ. 2023;16(2):209-223.

39. Wang, Y., Lv, X. 日常生活自理能力情景图示评定方法的设计[Design of pictorial assessment method for self-care ability in daily life]. Chin. j. of. Phys. Med. and. Rehabilit. 2018; 40 (11), 4.

40. Wang, Y., Ni, J., Sun, Z. 微信平台在开放性肘关节僵硬松解术后康复锻炼中的应用[Application of wechat platform in rehabilitation exercise after open elbow joint stiffness release]. International. J. Orthopaedics. 2018; 39 (06), 394-396.

41. Sun Z, Cui H, Ruan J, Li J, Wang W, Fan C. What Range of Motion and Functional Results Can Be Expected After Open Arthrolysis with Hinged External Fixation For Severe Post traumatic Elbow Stiffness? Clin Orthop Relat Res. 2019;477(10):2319-2328.

42. Sun Z, Li J, Luo G, Wang F, Hu Y, Fan C. What constitutes a clinically important change in Mayo Elbow Performance Index and range of movement after open elbow arthrolysis? Bone Joint J. 2021;103-B(2):366-372.

43. He X, Fen Q, Yang J, Lei Y, Heng L, Zhang K. Risk Factors of Elbow Stiffness After Open Reduction and Internal Fixation of the Terrible Triad of the Elbow Joint. Orthop Surg. 2021;13(2):530-536.

44. Zhang Y, Xia T, Huang L, Yin M, Sun M, Huang J, et al. Factors influencing user engagement of health information disseminated by chinese provincial centers for disease control and prevention on WeChat: observational study. JMIR Mhealth Uhealth. (2019) 7:e12245.

1. Bao Y, Wang C, Xu H, Lai Y, Yan Y, Ma Y, Yu T, Wu Y. Effects of an mHealth Intervention for Pulmonary Tuberculosis Self-management Based on the Integrated Theory of Health Behavior Change: Randomized Controlled Trial. JMIR Public Health Surveill. 2022 14;8(7):e34277.
2. Cheng C, Espanha R. Social support and audience engagement of lupus-related posts on social networking sites in China. Heliyon. 2024;10(11):e31754.
3. España K, Perris GE, Ngo NT, Bath E. Reimagining Narrative Approaches Through Comics for Systems-Involved Youth. J Am Acad Child Adolesc Psychiatry. 2024;63(8):766-770.
4. Castro-Alonso JC, de Koning BB, Fiorella L, Paas F. Five Strategies for Optimizing Instructional Materials: Instructor- and Learner-Managed Cognitive Load. Educ Psychol Rev. 2021;33(4):1379-1407.

49. Alemany-Pagès M, Azul AM, Ramalho-Santos J. The use of comics to promote health awareness: A template using nonalcoholic fatty liver disease. Eur J Clin Invest. 2022;52(3):e13642.

1. Cohn N. Your Brain on Comics: A Cognitive Model of Visual Narrative Comprehension. Top Cogn Sci. 2020;12(1):352-386.
2. Chen M, Bell RA. A meta-analysis of the impact of point of view on narrative processing and persuasion in health messaging. Psychol Health. 2022;37(5):545-562.
3. Wang Z, Romat H, Chevalier F, Riche NH, Murray-Rust D, Bach B. Interactive Data Comics. IEEE Trans Vis Comput Graph. 2022;28(1):944-954.
4. Walker A, Hing W, Lorimer A. The Influence, Barriers to and Facilitators of Anterior Cruciate Ligament Rehabilitation Adherence and Participation: a Scoping Review. Sports Med Open. 2020; 6(1):32-53.
5. Ntoumanis N, Ng JYY, Prestwich A, Quested E, Hancox JE, Thøgersen-Ntoumani C, et al. A meta-analysis of self-determination theory-informed intervention studies in the health domain: effects on motivation, health behavior, physical, and psychological health. Health Psychol Rev. 2021;15(2):214-244.
6. He S, Jiang S, Zhu R, Hu X. The influence of educational and emotional support on e-learning acceptance: An integration of social support theory and TAM. Educ Inf Technol (Dordr). Published online February 14, 2023.
