# Supplementary material for: Effectiveness of WeChat-Based Plus Scene-Graphics Health Education for Rehabilitation After Open Elbow Arthrolysis: Historical Control Study
Source: JMIR Form Res. 2025 Oct 21;9:e58218. doi: 10.2196/58218 (PMC12605337; doi:10.2196/58218)
Supplement: Multimedia Appendix 2 [file formative-v9-e58218-s002.docx]

W


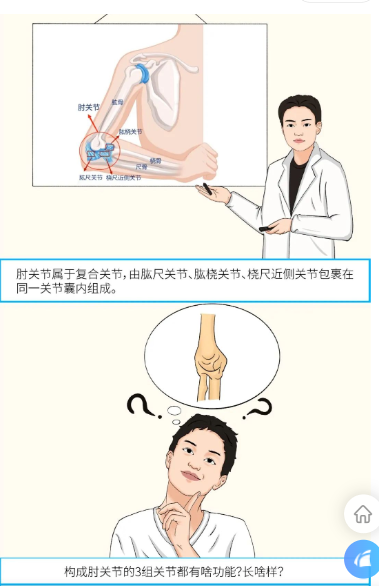

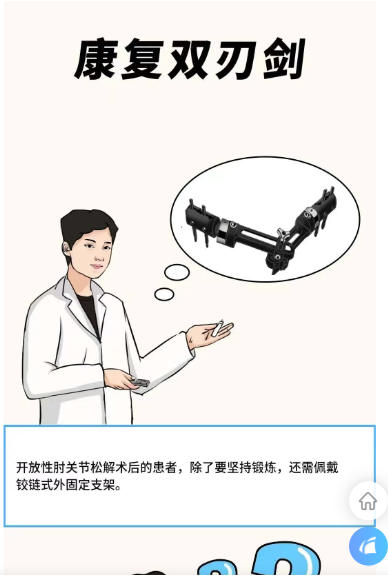


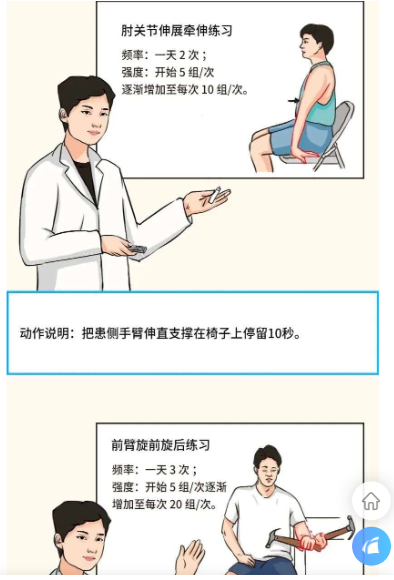

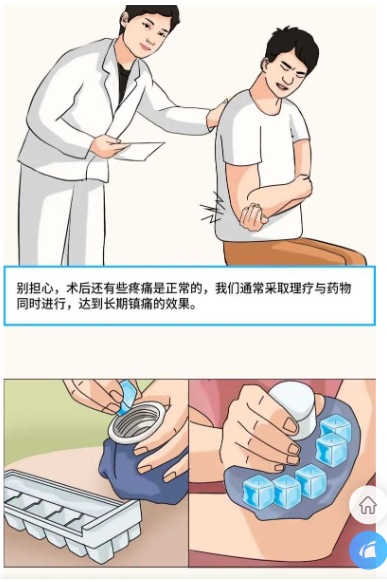


**Initial Elbow Joint Recognition**

**Rehabilitation: Double - edged**

**The song of R****ehabilitation**

**Advance despite hardship**

The materials of WeChat group


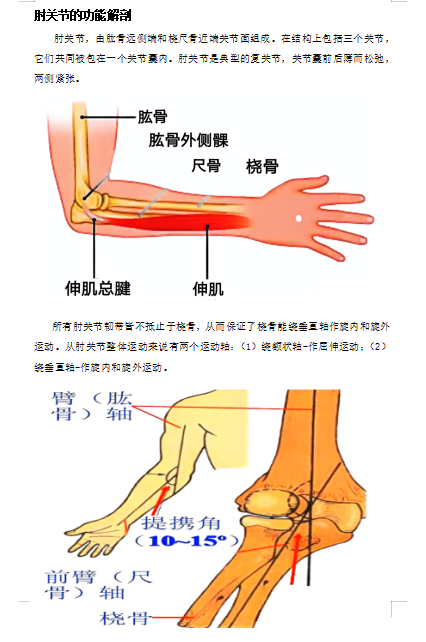

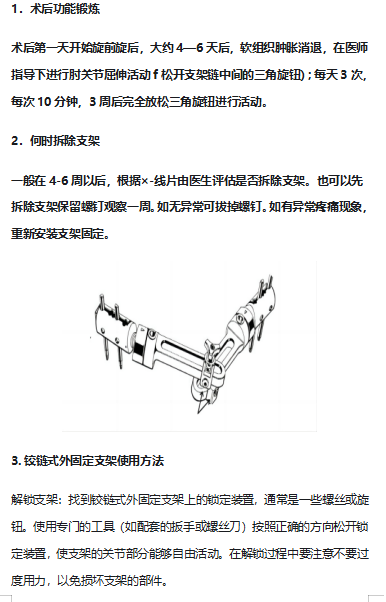


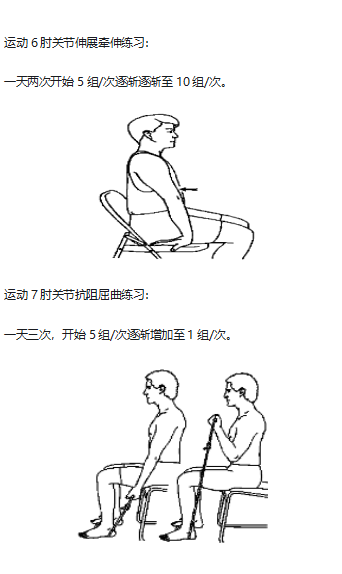

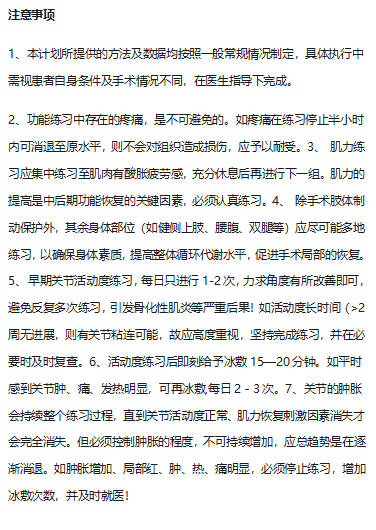


**Initial Elbow Joint Recognition**

**Rehabilitation: Double - edged**

**The song of Rehabilitation**

**Advance despite hardship**

The materials of Control group


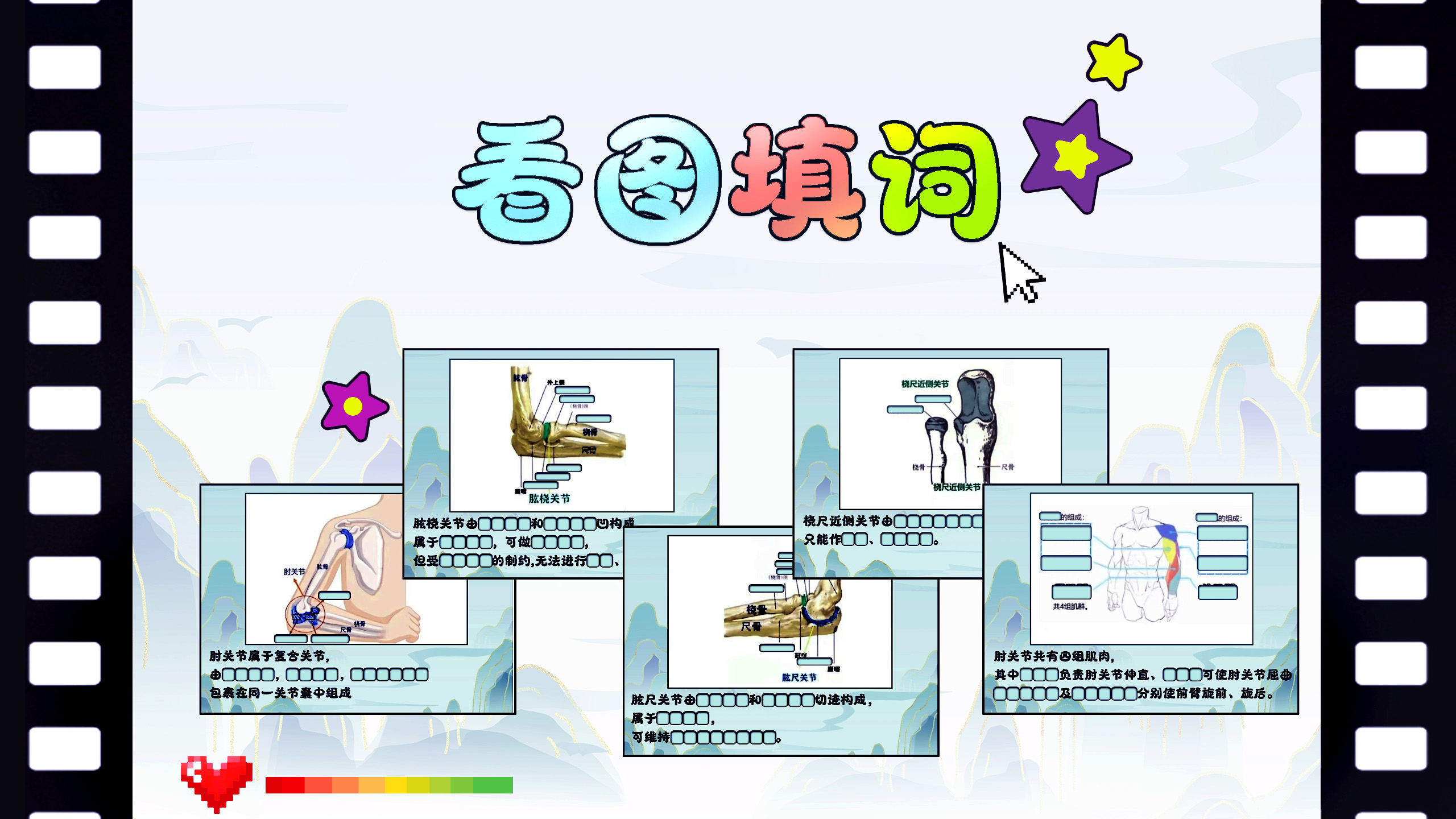


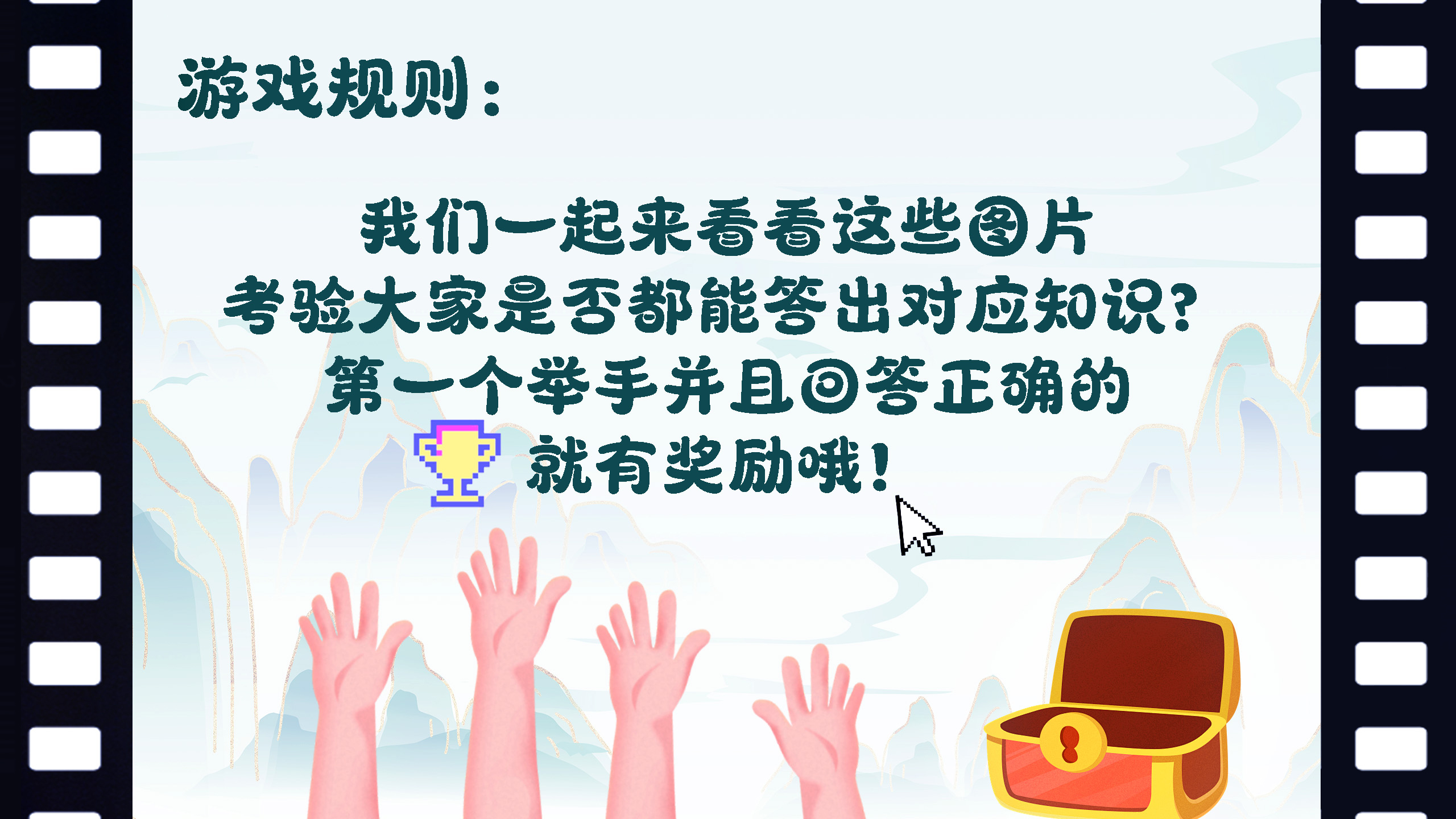


The material of Picture-based cloze test.


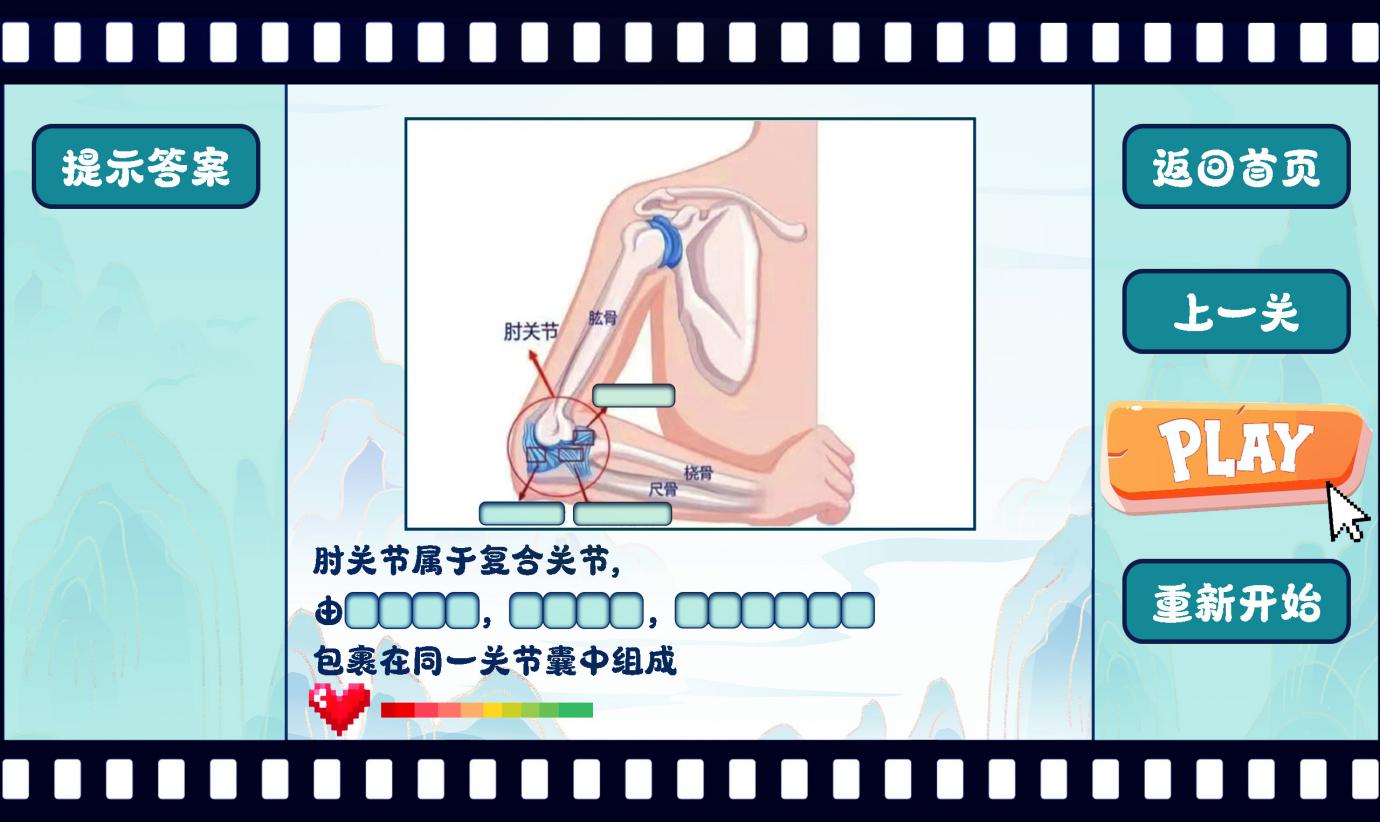


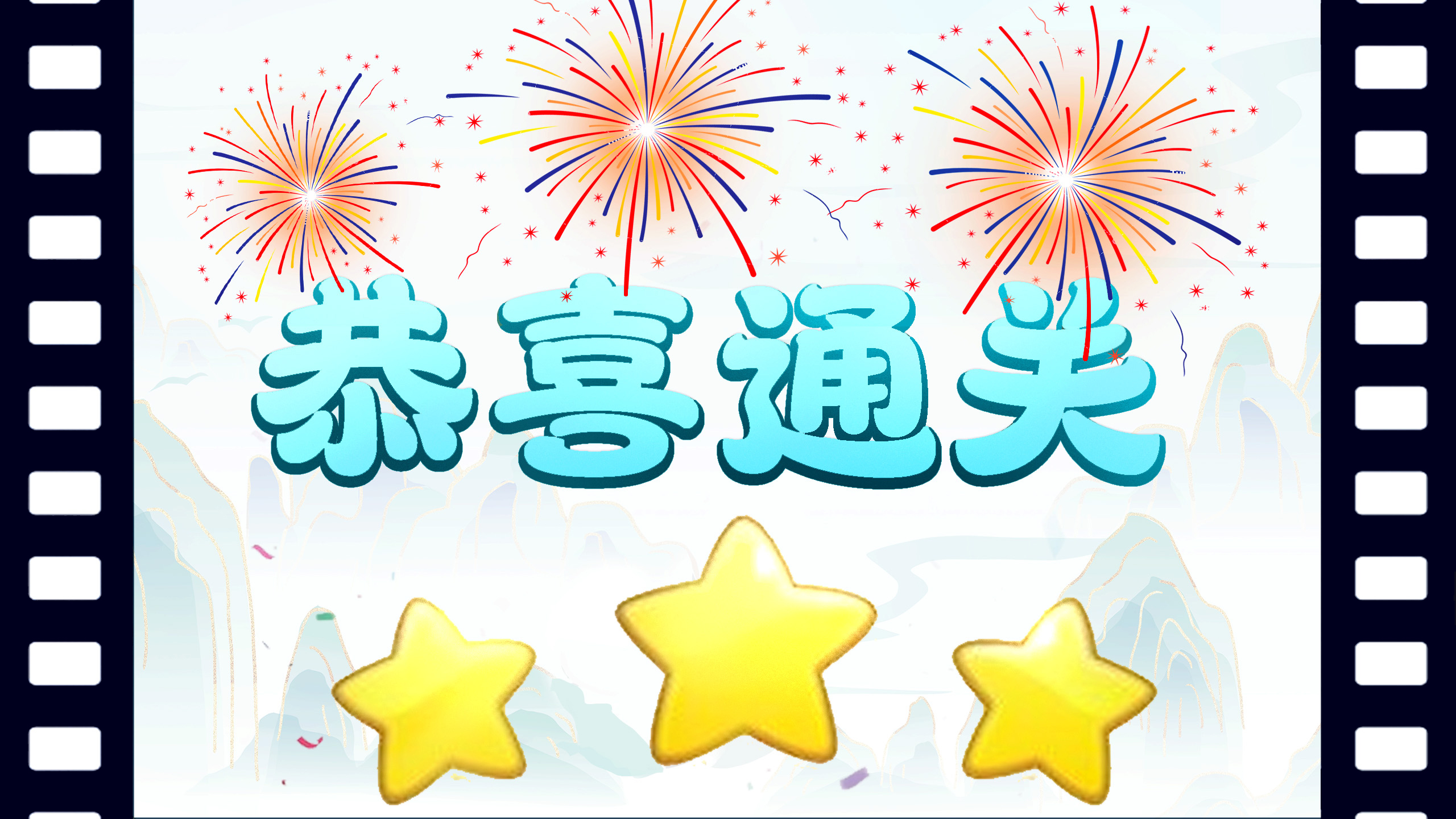


The material of Picture-based cloze test.

| ID | Send video daily? (Yes/No) | Days of sending videos per week | Check WeChat group messages daily? (Yes/No) | Total number of WeChat messages sent | WeChat group topic discussion  participation times | Time of first joining | Time of last being active |
| --- | --- | --- | --- | --- | --- | --- | --- |
| 001 | Yes | 7、7、7、7、7、7、7、7、7、7、7、7 | Yes | 350 | 30 | 2021 - 07 - 01 | 2021- 09 - 23 |
| 002 | Yes | 7、7、7、7、7、7、7、7、7、7、7、7 | Yes | 300 | 25 | 2021 - 07 - 01 | 2021- 09 - 23 |
| 003 | No | 7、7、6、7、6、7、7、7、7、7、7、7 | No | 180 | 15 | 2021 - 07 - 01 | 2021- 09 - 23 |
| 004 | Yes | 7、7、7、7、7、7、7、7、7、7、7、7 | Yes | 220 | 20 | 2021 - 07 - 01 | 2021- 09 - 23 |
| 005 | Yes | 7、7、7、7、7、7、7、7、7、7、7、7 | Yes | 260 | 28 | 2021 - 07 - 15 | 2021 - 10 -07 |
| 006 | Yes | 7、7、7、7、7、7、7、7、7、7、7、7 | Yes | 200 | 18 | 2021 - 07 - 15 | 2021 - 10 -07 |
| 007 | Yes | 7、7、7、7、7、7、7、7、7、7、7、7 | Yes | 380 | 32 | 2021 - 07 - 15 | 2021 - 10 -07 |
| 008 | No | 7、7、6、7、6、7、7、7、6、7、7、7 | No | 160 | 12 | 2021 - 07 - 15 | 2021 - 10 -07 |
| 009 | Yes | 7、7、7、7、6、7、7、7、7、7、7、7 | Yes | 240 | 22 | 2021 - 07 - 29 | 2021 - 10 - 21 |
| 010 | Yes | 7、7、7、7、7、7、7、7、7、7、7、7 | Yes | 290 | 27 | 2021 - 07 - 29 | 2021 - 10 - 21 |
| 011 | Yes | 7、7、7、7、7、7、7、7、7、7、7、7 | Yes | 220 | 20 | 2021 - 07 - 29 | 2021 - 10 - 21 |
| 012 | Yes | 7、7、7、7、7、7、7、7、7、7、7、7 | Yes | 360 | 33 | 2021 - 07 - 29 | 2021 - 10 - 21 |
| 013 | No | 7、7、6、7、6、7、6、7、7、7、7、7 | No | 190 | 16 | 2021 - 08 - 12 | 2021 - 11 - 04 |
| 014 | Yes | 7、7、7、7、6、7、7、7、7、7、7、7 | Yes | 250 | 23 | 2021 - 08 - 12 | 2021 - 11 - 04 |
| 015 | Yes | 7、7、7、7、7、7、7、7、7、7、7、7 | Yes | 280 | 26 | 2021 - 08 - 12 | 2021 - 11 - 04 |
| 016 | Yes | 7、7、7、7、7、7、7、7、7、7、7、7 | Yes | 210 | 19 | 2021 - 08 - 26 | 2021 - 11- 18 |
| 017 | Yes | 7、7、7、7、7、7、7、7、7、7、7、7 | Yes | 390 | 35 | 2021 - 08 - 26 | 2021 - 11- 18 |
| 018 | No | 7、7、7、7、6、7、7、7、7、6、7、7 | No | 150 | 13 | 2021 - 08 - 26 | 2021 - 11- 18 |
| 019 | Yes | 7、7、7、7、7、7、7、7、7、7、7、7 | Yes | 230 | 21 | 2021 - 08 - 26 | 2021 - 11- 18 |
| 020 | Yes | 7、7、7、7、7、7、7、7、7、7、7、7 | Yes | 270 | 25 | 2021 - 09 - 09 | 2021 - 12 - 02 |
| 021 | Yes | 7、7、7、7、7、7、7、7、7、7、7、7 | Yes | 200 | 18 | 2021 - 09 - 09 | 2021 - 12 - 02 |
| 022 | Yes | 7、7、7、7、7、7、7、7、7、7、7、7 | Yes | 370 | 34 | 2021 - 09 - 09 | 2021 - 12 - 02 |
| 023 | No | 7、7、7、7、6、7、7、6、7、7、7、7 | No | 170 | 15 | 2021 - 09 - 23 | 2021 - 12 - 16 |
| 024 | Yes | 7、7、7、7、7、7、7、7、7、7、7、7 | Yes | 260 | 24 | 2021 - 09 - 23 | 2021 - 12 - 16 |
| 025 | Yes | 7、7、7、7、7、7、7、7、7、7、7、7 | Yes | 300 | 28 | 2021 - 09 - 23 | 2021 - 12 - 16 |
| 026 | Yes | 7、7、7、7、7、7、7、7、7、7、7、7 | Yes | 230 | 21 | 2021 - 10 - 14 | 2022 - 01 - 06 |
| 027 | Yes | 7、7、7、7、7、7、7、7、7、7、7、7 | Yes | 400 | 36 | 2021 - 10 - 14 | 2022 - 01 - 06 |
| 028 | No | 7、7、7、7、6、7、7、7、7、6、7、7 | No | 180 | 16 | 2021 - 10 - 14 | 2022 - 01 - 06 |
| 029 | Yes | 7、7、7、7、7、7、7、7、7、7、7、7 | Yes | 250 | 23 | 2021 - 10 - 28 | 2022 - 01 - 20 |
| 030 | Yes | 7、7、7、7、7、7、7、7、7、7、7、7 | Yes | 310 | 29 | 2021 - 10 - 28 | 2022 - 01 - 20 |
| 031 | Yes | 7、7、7、7、7、7、7、7、7、7、7、7 | Yes | 220 | 20 | 2021 - 10 - 28 | 2022 - 01 - 20 |
| 032 | Yes | 7、7、7、7、7、7、7、7、7、7、7、7 | Yes | 380 | 35 | 2021-  11-18 | 2022 - 02 - 10 |
| 033 | No | 7、7、7、7、6、7、7、7、7、7、7、6 | No | 200 | 18 | 2021-  11-18 | 2022 - 02 - 10 |
| 034 | Yes | 7、7、7、7、7、7、7、7、7、7、7、7 | Yes | 270 | 25 | 2021-  11-18 | 2022 - 02 - 10 |
| 035 | Yes | 7、7、7、7、7、7、7、7、7、7、7、7 | Yes | 320 | 30 | 2021-  11-18 | 2022 - 02 - 10 |
| 036 | Yes | 7、7、7、7、7、7、7、7、7、7、7、7 | Yes | 240 | 22 | 2021-  11-25 | 2022 - 02 - 17 |
| 037 | Yes | 7、7、7、7、7、7、7、7、7、7、7、7 | Yes | 420 | 38 | 2021-  11-25 | 2022 - 02 - 17 |
| 038 | No | 7、7、6、7、6、7、7、7、7、6、7、6 | No | 160 | 14 | 2021-  11-25 | 2022 - 02 - 17 |
| 039 | Yes | 7、7、7、7、7、7、7、7、7、7、7、7 | Yes | 260 | 24 | 2021-  11-25 | 2022 - 02 - 17 |
| 040 | Yes | 7、7、7、7、7、7、7、7、7、7、7、7 | Yes | 300 | 28 | 2021-  12-09 | 2022-  03-03 |
| 041 | Yes | 7、7、7、7、7、7、7、7、7、7、7、7 | Yes | 230 | 21 | 2021-  12-09 | 2022-  03-03 |
| 042 | Yes | 7、7、7、7、7、7、7、7、7、7、7、7 | Yes | 390 | 36 | 2021-  12-09 | 2022-  03-03 |
| 043 | No | 7、7、7、7、6、7、7、7、7、7、7、5 | No | 190 | 17 | 2021-  12-09 | 2022-  03-03 |
| 044 | Yes | 7、7、7、7、7、7、7、7、7、7、7、7 | Yes | 280 | 26 | 2021-  12-16 | 2022-  03-10 |
| 045 | Yes | 7、7、7、7、7、7、7、7、7、7、7、7 | Yes | 330 | 31 | 2021-  12-16 | 2022-  03-10 |
| 046 | Yes | 7、7、7、7、7、7、7、7、7、7、7、7 | Yes | 250 | 23 | 2021-  12-16 | 2022-  03-10 |
| 047 | Yes | 7、7、7、7、7、7、7、7、7、7、7、7 | Yes | 430 | 39 | 2021-  12-16 | 2022-  03-10 |
| 048 | No | 7、7、7、7、5、7、7、7、7、7、6、6 | No | 170 | 15 | 2021-  12-16 | 2022-  03-10 |
| 049 | Yes | 7、7、7、7、7、7、7、7、7、7、7、7 | Yes | 270 | 25 | 2021-  12-23 | 2022-  03-17 |
| 050 | Yes | 7、7、7、7、7、7、7、7、7、7、7、7 | Yes | 310 | 29 | 2021-  12-23 | 2022-  03-17 |
| 051 | Yes | 7、7、7、7、7、7、7、7、7、7、7、7 | Yes | 240 | 22 | 2021-  12-23 | 2022-  03-17 |
| 052 | Yes | 7、7、7、7、7、7、7、7、7、7、7、7 | Yes | 400 | 37 | 2021-  12-23 | 2022-  03-17 |
| 053 | No | 7、7、7、7、6、7、7、7、5、7、6、7 | No | 210 | 19 | 2021-  12-30 | 2022-  03-24 |
| 054 | Yes | 7、7、7、7、7、7、7、7、7、7、7、7 | Yes | 290 | 27 | 2021-  12-30 | 2022-  03-24 |
| 055 | Yes | 7、7、7、7、7、7、7、7、7、7、7、7 | Yes | 340 | 32 | 2021-  12-30 | 2022-  03-24 |
| 056 | Yes | 7、7、7、7、7、7、7、7、7、7、7、7 | Yes | 260 | 24 | 2021-  12-30 | 2022-  03-24 |
